# Hierarchical porous silicon structures with extraordinary mechanical strength as high-performance lithium-ion battery anodes

**DOI:** 10.1038/s41467-020-15217-9

**Published:** 2020-03-19

**Authors:** Haiping Jia, Xiaolin Li, Junhua Song, Xin Zhang, Langli Luo, Yang He, Binsong Li, Yun Cai, Shenyang Hu, Xingcheng Xiao, Chongmin Wang, Kevin M. Rosso, Ran Yi, Rajankumar Patel, Ji-Guang Zhang

**Affiliations:** 1 Energy and Environment Directorate, Pacific Northwest National Laboratory, Richland, Washington 99352 USA; 2 Physical and Computational Sciences Directorate, Pacific Northwest National Laboratory, Richland, Washington 99352 USA; 3 Environmental Molecular Sciences Laboratory, Pacific Northwest National Laboratory, 3335 Innovation Boulevard, Richland, Washington 99354 USA; 4 General Motors Research and Development Center, 30500 Mound Road, Warren, Michigan 48090 USA; 5 National Security Directorate, Pacific Northwest National Laboratory, Richland, Washington 99352 USA

**Keywords:** Chemistry, Electrochemistry, Batteries

## Abstract

Porous structured silicon has been regarded as a promising candidate to overcome pulverization of silicon-based anodes. However, poor mechanical strength of these porous particles has limited their volumetric energy density towards practical applications. Here we design and synthesize hierarchical carbon-nanotube@silicon@carbon microspheres with both high porosity and extraordinary mechanical strength (>200 MPa) and a low apparent particle expansion of ~40% upon full lithiation. The composite electrodes of carbon-nanotube@silicon@carbon-graphite with a practical loading (3 mAh cm^−2^) deliver ~750 mAh g^−1^ specific capacity, <20% initial swelling at 100% state-of-charge, and ~92% capacity retention over 500 cycles. Calendered electrodes achieve ~980 mAh cm^−3^ volumetric capacity density and <50% end-of-life swell after 120 cycles. Full cells with LiNi_1/3_Mn_1/3_Co_1/3_O_2_ cathodes demonstrate >92% capacity retention over 500 cycles. This work is a leap in silicon anode development and provides insights into the design of electrode materials for other batteries.

## Abstract (web summary)

The authors here construct hierarchical porous CNT@Si@C microspheres as anodes for Li-ion batteries, enabling both high electrochemical performance and excellent mechanical strength. The work highlights the importance of mechanical properties in developing battery materials for practical applications.

## Introduction

As batteries become increasingly indispensable to the daily life of human society with their expanded use from cell phones and portable electronics to electric vehicles and electric grids, the demands on batteries with even higher energy densities are continuously increasing^1–4^. The use of nanostructured materials has advanced battery technologies, particularly the development of high-performance and low-cost lithium-ion batteries (LIBs) and beyond^5–11^. The development of silicon (Si) anodes for next-generation LIBs has benefited tremendously from the introduction of nanotechnology. In the past decade, landmark progress has been achieved in promoting the electrochemical performance of Si anodes using Si-based nanostructures including Si nanoparticles, nanowires, nanotubes, yolk-shell structures, etc^12–26^. Our group also has developed mesoporous Si sponge and designed porous Si/graphite (Gr) composite anodes to mitigate pulverization and reduce particle swelling, hence achieving high-performance anodes at practical loadings^27,28^. An et al.^29^ recently reported an ant-nest-like bulk porous Si, which demonstrates impressive performance in both half-cell and full-cell configurations.

Although nanostructured Si can mitigate its structure failure originated from large volume change during lithiation/delithiation processes, the properties intrinsic to nanomaterials such as high surface area and low tap density are also detrimental for their electrochemical performances and the manufacturing for practical batteries. For example, the nanostructured Si materials with a large surface area often have a poor adhesion to the current collector at high loading (>3 mAh cm^−2^) and more serious parasitic electrolyte decomposition reactions lead to lower first cycle Coulombic efficiency (CE)^23,25^. Efforts on binder, electrolyte, surface coating, and prelithiation are needed to improve the electrode integrity, solid electrolyte interphase (SEI) residency and lithium loss in full cells using Si anodes^30–37^.

Furthermore, many porous nanostructure Si particles have poor mechanical strength and are easy to be broken during calendering, which is a critical/must step in practical battery manufacturing to reduce the parasitic reactions, electrolyte consumption, and improve battery energy density and safety. Recently, it has been realized that the excellent mechanical strength is as essential as superb electrochemical performance for nanostructured battery materials^38–40^. Yolk-shell Si@titanium dioxide with improved mechanical strength and Si-nanolayer-embedded Gr can be calendered to reach high-capacity density^17,41^. However, it is still an onerous quest to design nanostructures with satisfactory mechanical and electrochemical performance for battery applications.

Carbon nanotubes (CNTs), which are known for high electronic conductivity and excellent mechanical properties^42–44^, have been harnessed into composites with nano-Si to improve the cycle life of Si anodes^45–47^. In this work, unique hierarchical porous CNT@Si@carbon (CNT@Si@C) microspheres of high mechanical strength and limited particle swelling upon full lithiation are developed with well-engineered structural parameters (small primary Si particle size, controlled porosity, and surface area, high-quality carbon coating, etc). CNT@Si microspheres are prepared by in-situ thermite reduction of microspheres of CNT@silica (CNT@SiO_2_) core-shell coaxial cables. The well-designed CNT@Si microspheres can absorb the volume change of Si and hence demonstrate apparent particle expansion of ~30% upon full lithiation, which is 1/10 of the expansion of bulk Si particles. The yarn-ball-like CNT@Si microspheres after carbon coating, denoted as CNT@Si@C, has ~40% particle expansion upon full lithiation. It also can withstand >200 MPa pressure without breakdown and therefore can tolerate the industrial calendering process for electrode manufacturing. With this unique structure, the CNT@Si@C anode delivers a reversible capacity of ~1500 mAh g^−1^ and 87% capacity retention over 1500 cycles at 1 mA cm^−2^. The practical CNT@Si@C-Gr composite anodes at 3 mAh cm^−2^ areal loading exhibit ~750 mAh g^−1^ specific capacity, <20% initial swelling at 100% state-of-charge, and ~92% capacity retention over 500 cycles. The calendered electrodes demonstrate ~980 mAh cc^−3^ volumetric capacity density and <50% end-of-life (EOL) swell after 120 cycles. The full cell with LiNi_1/3_Mn_1/3_Co_1/3_O_2_ cathode demonstrates >92% capacity retention over 500 cycles. Our work represents an effective strategy in the development of Si anodes for LIBs and possibly other battery chemistry with fundamental understanding of the electro-mechanical stability of porous Si.

## Results

### Structural design of hierarchical CNT@Si@C microspheres

**Fig. 1:**
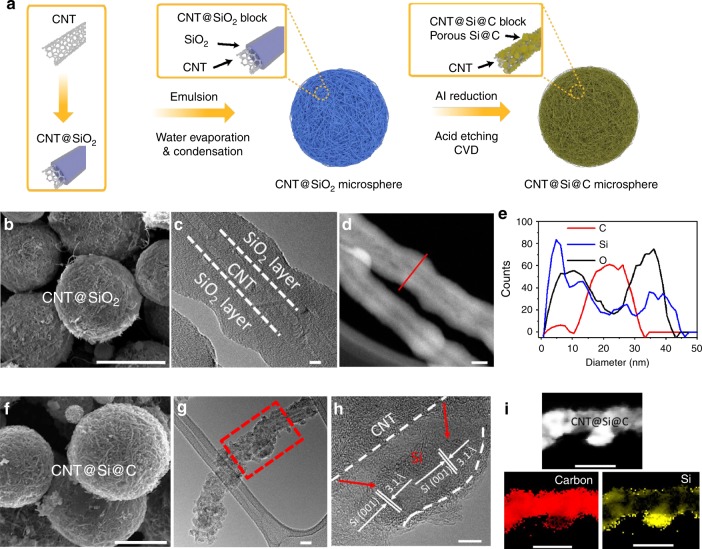
Schematic of synthesis process and structural characterization of the key intermediate products of CNT@SiO_2_ and final CNT@Si@C microspheres. **a** Schematic figure showing the synthesis of CNT@Si@C microspheres. **b** A typical SEM image of CNT@SiO_2_ microspheres (scale bar = 5 µm). **c** A representative TEM image of a CNT@SiO_2_ core-shell coaxial cable (scale bar = 5 µm). **d** A STEM image of the CNT@SiO_2_ cables (scale bar = 20 µm). **e** EDS line scan of the CNT@SiO_2_ cable marked in **d**. Small discrepancy of Si and O due to beam effect from prolonged scanning. **f** A typical SEM image of CNT@Si@C microspheres (scale bar = 3 µm). **g** Low-magnification TEM image of a composite cable from a CNT@Si@C microsphere (scale bar = 20 µm), **h** HRTEM image of the cable in **g** (scale bar = 5 µm). **i** EDS mapping of the marked area in **g** (scale bar = 50 µm).

Figure 1 shows the schematic synthesis process (Fig. 1a) and structure characterization of the key intermediate products of CNT@SiO_2_ (Fig. 1b–e) and the final carbon-coated hierarchical porous CNT@Si@C microspheres (Fig. 1f–i). The CNT@SiO_2_ microspheres (Fig. 1b) were prepared by emulsion of the CNT@SiO_2_ core-shell coaxial cables (Fig. 1c), which was prepared with a sol-gel method^48^. The SiO_2_ layer of ~10–20 nm thick was uniformly coated on the surface functionalized high-quality CNTs (Supplementary Fig. 1, outer diameter 20–40 nm) and formed core-shell coaxial cables of ~60–80 nm (Fig. 1c and Supplementary Fig. 2). Scanning transmission electron microscopy (STEM) and energy-dispersive X-ray spectroscopy (EDS) line scan (Fig. 1d–e) corroborated the coating uniformity with slightly varying thickness. The SiO_2_ coating was designed by controlling the tetraethoxysilane (TEOS) percentage so that the final CNT@Si@C anodes have a target-specific capacity of ~1000 mAh g^−1^ based on the total electrode weight (see Supplementary Fig. 3 and design detail in Supplementary Materials).

Because of the unique structure design, the CNT@SiO_2_ microspheres have uniformly distributed Si, oxygen, and carbon (Supplementary Fig. 4) even though the particle is ~6–8 µm large in diameter (Supplementary Fig. 5). Furthermore, the CNT@Si microspheres produced from the CNT@SiO_2_ microspheres by aluminothermic reduction maintain the spherical morphology and similar size (Supplementary Fig. 6). The Si and carbon also are uniformly distributed in the CNT@Si microspheres (Supplementary Fig. 6). The formation of Si was corroborated by the X-ray diffraction (XRD) (Supplementary Fig. 7) and further characterization of the final product of CNT@Si@C (Fig. 1f–i). No signal associated to Al is detected by X-ray photoelectron spectroscopy (XPS) for CNT@Si, reflecting the complete removal of Al during the washing steps (Supplementary Fig. 8).

CNT@Si was coated with a layer of carbon using chemical vapor deposition (CVD) method to help prevent the oxidization of nano-Si in air and control the surface area, and hence the electrochemical performance (vide infra). CVD carbon, even though is not as robust as pitch carbon^49^, has the advantage in easy pore filling and forming thin and uniform carbon coating on as much surface of the porous Si as possible. CNT@Si@C has reduced surface area and porosity (Supplementary Figs. 9–11 and Supplementary Table 1) and thus results in reduced parasitic reaction from electrolyte decomposition and improved electrochemical performance. The tap density of the CNT@Si@C is 0.5 g cc^−1^, more than three times larger than the tap density of nano-Si powder (~0.15 g cc^−1^)^19^. XPS analyses (Supplementary Fig. 12) showed ~6–8 wt% of oxygen in CNT@Si and CNT@Si@C, similar to the EDS measurement results (Supplementary Fig. 13, 7.3–8.5 wt%). Supplementary Fig. 14 is Raman spectra showing high-quality carbon with similar G/D ratio to that of the CNT was infiltrated into the pores of hierarchical porous CNT@Si and coated on the surface. TEM and EDS mapping show the detailed composite structure of CNT@Si@C (Fig. 1g–i). Figure 1 and Supplementary Fig. 15 show that Si particles with various sizes up to tens of nanometers were decorated on the CNT. XRD analysis shows that Si has an average crystallite size of ~30–40 nm, which is consistent with the distribution histogram from TEM (Supplementary Fig. 16). High-resolution (HR) TEM (Fig. 1h) reveals the crystalline structure of some large nanoparticles with lattice fringes of 3.1 Å, corresponding to Si (111) plane, while the mapping shows some amorphous Si or fine clusters. The carbon layer of ~4 nm thick uniformly coats on Si nanoparticles (Fig. 1h–i and Supplementary Fig. 15). Fourier-transform infrared spectroscopy study (Supplementary Fig. 17) show the chemical binding between Si, CNT, or CVD carbon coating. The improved mechanical or electrical integrity is due to the chemical bonding between CNT, Si, and C, as well as the physical attachment and CVD carbon-coating effect.

**Fig. 2:**
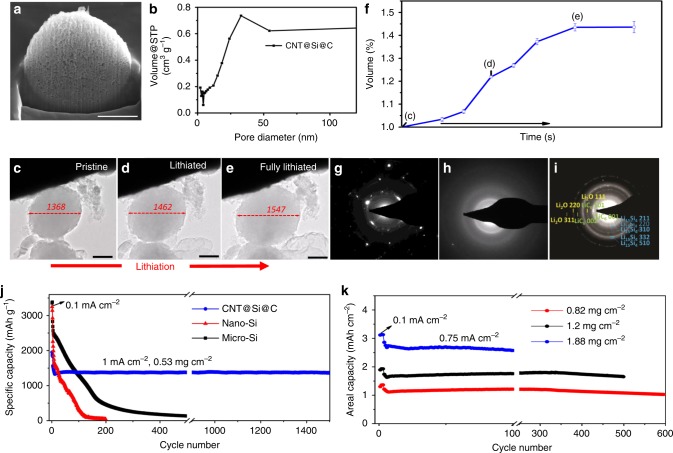
Characterization of the CNT@Si@C porous structure, particle swelling, and electrochemical performance. **a** Cross-section SEM of a typical CNT@Si@C microsphere (scale bar = 1 µm). **b** Pore diameter distribution of CNT@Si@C from gas absorption. **c**–**e** In-situ TEM images of a CNT@Si@C particle at different lithiation states (scale bar = 0.5 µm). The unit of the numbers in figures is nm. **f** Volume expansion curve of the particle recorded during the lithiation process. **g**–**h** The selected area electron diffraction (SAED) patterns of CNT@Si@C at different lithiation states. **j** Long-term cycling of CNT@Si@C, nano-Si, and micro-Si. **k** Long-term cycling of CNT@Si@C at different mass loadings.

A porous structure is essential to accommodate the volume expansion of Si, yet it needs to be well-tailored to retain sufficient volumetric capacity density and high mechanical strength that can withstand calendering and other battery assembly procedures. The porous structure evolution from CNT@SiO_2_ microsphere to CNT@Si and CNT@Si@C were monitored by cross-section scanning electron microscopy (SEM) and gas absorption (Fig. 2 and Supplementary Figs. 9–11). The SEM study showed improved pore distribution and porous structure for CNT@Si compared with CNT@SiO_2_. The gas absorption showed the surface area, pore volume, and average pore diameter increase from CNT@SiO_2_ to CNT@Si, whereas these parameters decrease for CNT@Si@C (Supplementary Table 1). The final carbon-coated CNT@Si microspheres, CNT@Si@C, have a surface area (~60 m^2^ g^−1^) smaller than many nano-Si materials, while still having an average pore diameter of 20 nm to accommodate the expansion of Si nanoparticles anchored on CNTs.

The effects of the porous structure of CNT@Si and CNT@Si@C in accommodating the Si volume expansion were investigated by in-situ particle swelling measurements and electrochemical tests. With the unique highly porous yarn-ball-like structure, both CNT@Si and CNT@Si@C microspheres deliver the expected effect on suppressing the swelling of porous Si particles upon lithiation, while maintaining overall structural integrity. In-situ TEM characterization of a representative CNT@Si microsphere with ~1.12 µm in diameter expanded to a sphere of ~1.14 µm at partial lithiation and to ~1.21 µm at full lithiation (Supplementary Fig. 18a–c and Supplementary Movie 1). The three-dimensional (3D) expansion calculated from the sphere volume is ~27% and no cracking was observed. The hierarchical porous structure is effective in mitigating the swelling and maintaining structural integrity. Multiple particles were similarly checked and the average structure swelling is ~30%, which is 1/10 the expansion of bulk Si particles (Supplementary Fig. 18d–f and Supplementary Movie 2). In case of CNT@Si@C, the in-situ particle swelling measurement showed that a typical CNT@Si@C sphere at full lithiation has a volume expansion of ~44% (Fig. 2c–f and Supplementary Movie 3). Selected area electron diffraction patterns during the lithiation have shown that the porous Si changed from polycrystalline (Fig. 2g) to amorphous (Fig. 2h) and then to crystalline Li_15_Si_4_ phase after full lithiation (Fig. 2i). The increased volume expansion of CNT@Si@C is due to the decrease of pore size and pore volume after carbon coating (Supplementary Table 1). In-situ TEM (Supplementary Fig. 19) also showed that the size of Si particles increases as the lithiation process goes on, while no fracture was observed even after full lithiation. The particles demonstrate good flexibility throughout the lithiation process and the bonding between CNT and Si remains good during the repeated cycling (see Supplementary Movie 4).

The electrochemical performance of CNT@Si@C in a side-by-side comparison with nano-Si or micron-Si as shown in Fig. 2j and Fig. 2k, respectively, at various electrode loadings provides the final and best proof of having achieved the desired porous structure. Figure 2j showed much better cycling stability of the CNT@Si@C than nano-Si or micron-Si at similar low electrode loading (0.75 mAh cm^−2^). CNT@Si@C showed almost no fading after the formation cycles. The capacity retention is >87% (compared with the first cycle after formation) over ~1500 cycles. Control experiments using conventional nanostructured CNT-Si-carbon composites and CNT@Si microsphere anodes were conducted as well. The nanostructured CNT-Si-carbon composites present a fast capacity fade because of poor adhesion between the electrodes and current collectors, whereas the CNT@Si microspheres demonstrate an initial capacity of 2441 mAh g^−1^ with 72% capacity retention after 240 cycles (Supplementary Fig. 20). The above side-to-side comparison further confirms the advantage of the yarn-ball-like CNT@Si@C microsphere design and the importance of the carbon coating. Figure 2k demonstrates that the CNT@Si@C electrode can still deliver excellent cyclability at increased electrode loadings. The electrode with a practical loading of ~3 mAh cm^−2^ still retains ~90% capacity after 100 cycles.

Detailed analysis of the battery performance further corroborated the success of our initial structure design. First, the first delithiation capacity of the CNT@Si@C electrode is ~1900 mAh g^−1^ at low current density of 0.1 mA cm^−2^ and ~1500 mAh g^−1^ at 1 mA cm^−2^ (Fig. 2j and Supplementary Fig. 21). With ~14 wt% CNT and 20 wt% of CVD carbon (Thermogravimetric analysis in Supplementary Fig. 22), the Si in the composite has ~2850 mAh g^−1^ specific capacity; this is almost the delithiation capacity of commercial nano-Si, corroborating the structural design (Supplementary Fig. 3). Second, postmodern analysis of the electrodes showed that the CNT@Si@C microspheres preserved the original morphology even after 500 deep cycles (Supplementary Fig. 23). Third, the average thickness of pristine CNT@Si@C is 42.5 µm, which increases to 53 µm after initial lithiation (Supplementary Figs. 24–26). Thus, the CNT@Si@C electrode swelling at 100% state-of-charge (SOC) is ~24.7% [(53 − 42.5)/42.5]. In comparison with the particle swelling from in-situ TEM, the smaller electrode swelling at the electrode level is ascribed to the additional porosity in the electrode. The SEI formation, which is known to lead to the increase of electrode thickness, was not severe for the CNT@Si@C microsphere anodes. Last, the CNT@Si@C-NMC333 full cells demonstrate good cycling stability with >91.7% capacity retention over 100 cycles (Supplementary Fig. 27).

### Mechanical strength of the CNT@Si@C microspheres

**Fig. 3:**
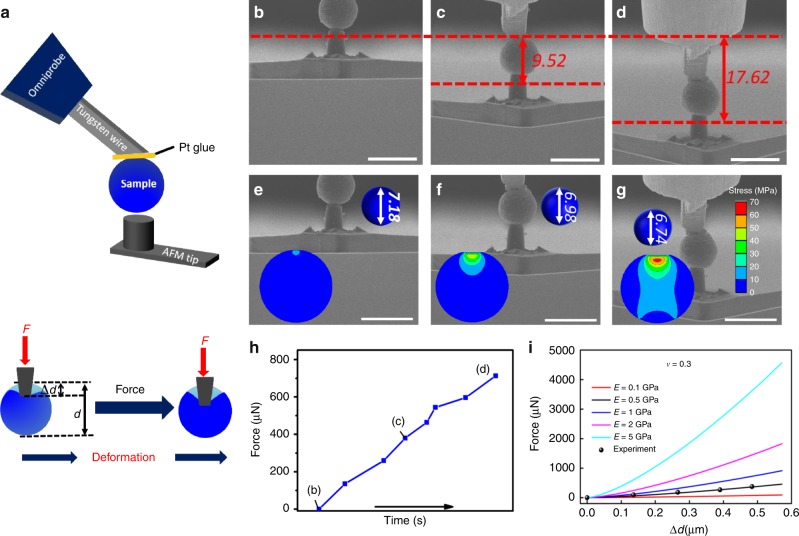
Mechanical strength of CNT@Si@C microspheres. **a** Schematic of the in-situ AFM–SEM experiment for the mechanical strength measurement. **b**–**d** AFM tip displacement/cantilever deflection of a typical CNT@Si@C sphere under an applied force: **b** beginning of the pressing; **c** the middle point of the pressing; **d** right before the breakdown of the particle. **e**–**g** The different deformation states of a CNT@Si@C sphere: **e** ∆*d* = 0.05 µm; **f** ∆*d* = 0.27 µm; **g** ∆*d* = 0.49 µm, and the corresponding distribution of the von Mises stress (MPa) from FE simulation (inset) with the Young’s modulus 0.5 GPa and the Poisson ratio 0.3. Scale bar for Fig. 3b–g is 10 µm. The unit for the numbers in the figures is µm. **h** The curve of force vs time during **b** to **d**. **i** Experimental data (dots) and FE simulation curve of the force vs particle deformation Δ*d* with the Poisson ratio 0.3 and different Young’s modulus.

The mechanical strength of CNT@Si@C was measured by in-situ atomic force microscopy (AFM) and SEM experiments. Figure 3a shows the experimental schematic of pressing an individual particle against an AFM tip with appropriate spring constant. A single microsphere was welded to the rigid W probe, which is connected to an OMNI probe. The microsphere and the W probe have no relative movement and moves slowly as a whole to press a fixed AFM tip. The force on the AFM tip and the deformation of the microspheres were recorded in-situ to assess the Young’s modulus, Poisson ratio, and mechanical strength of the CNT@Si@C. Supplementary Fig. 28 shows the typical fabrication process of the AFM tip for this mechanical strength measurement. Knowing the diameter and area of the AFM tip is as important as knowing the spring constant for accurate calculation of the resistive force. Figure 3b–d were recorded at the beginning, in the middle, and before breakage of a microsphere with a diameter of ~7.23 μm (see the pressing process in Supplementary Movie 5 and Supplementary Fig. 28). The AFM tip displacement/cantilever deflection and the deformation of the microsphere (∆*d*) were carefully measured (see the results in Supplementary Figs. 29 and 30). The compressive force applied on the particle, which is calculated from the AFM tip displacement/cantilever deflection and spring constant, is plotted in Fig. 3h. The results in Fig. 3h and Supplementary Fig. 29 show that the particle can withstand a total force of 705 µN or an equivalent pressure of ~181 MPa before breakage. Similar mechanical measurements were systematically investigated on particles of different sizes and using different AFM tips to produce reliable results and minimize the sample/measurement variation (Supplementary Fig. 31 and Supplementary Movie 6). From these results, we can see clearly that the CNT@Si@C has remarkable mechanical strength that can withstand ~200 MPa pressure without cracking or serious deformation. Considering the calculations were from the moments right before the fracture of the microsphere happened, the force has not reached the fracture point yet it already corresponds to ~200 MPa pressure, demonstrating high mechanical strength of our hierarchical porous microspheres.

To assess the Young’s modulus, a finite element (FE) method was used to simulate the deformation of the microsphere with exactly the same boundary conditions as that in the in-situ AFM–SEM experiments, i.e., fixed displacement constraint on the welded area, given deformation (∆*d*) boundary condition on the contacted area between the microsphere and AFM tip, and stress-free boundary condition on the rest surface of the microsphere. The von Mises stress distributions for the sequence of different deformation states are shown in Fig. 3e-g. The von Mises stress beneath the pressing tip is much larger than that near the bottom due to the different boundary conditions at the bottom and top of the particle. The high von Mises stress should cause breaks near the pressing tip, as visualized in Supplementary Movie S6. The curves of the force vs. time and the deformation of the particle (Δ*d*) calculated from FEM simulations for different elastic properties are plotted in Fig. 3h–i. Comparing the simulation and experimental results, the Young’s modulus and Poisson ratio are determined to be 0.5 GPa and 0.3, respectively. The model settings and simulation results are presented in detail in the Supplementary Materials (Supplementary Figs. 32 and 33, and Supplementary Movie 7).

A flat punch experiment (the substrate and the probe are two infinite planes in comparison with the microsphere) using freestanding microsphere also was carried out for further evaluation of the mechanical strength of the CNT@Si@C material (Supplementary Figs. 34 and 35). The young’s modulus measured from the flat punch experiment is ~0.5–0.8 GPa, which matches well with the result from in-situ AFM–SEM measurements and FE simulations. It supports that the results from our mechanical measurements and FE simulations are reliable. The stress at the end of the elastic deformation in the flat punch experiment is ~48.4 MPa, whereas it is ~91.2 MPa at the end of densification. The microsphere mechanical strength from the flat punch experiments is between the two numbers but smaller than the value measured by in-situ AFM–SEM design because of different experiment settings and boundary conditions (the displacement constraint in the in-situ AFM–SEM experiments should reduce the deformation and result in increased fracture strength). It must be noted that the elastic deformation strength of 48.4 MPa is still larger than the facture strength of core-shell structures^17^ and maybe other porous structures. It is believed that the high mechanical strength of the CNT@Si@C is from the superior properties of CNT and the microsphere yarn-ball-like matrix structure. CNT is known to have high Young’s modulus values between 12 and 50 GPa, and is hard to be compressed axially or radially^42,50^. In addition, the CNT yarn ball may yield slightly and lose some porosity under very high compressing force, but it will not break. The appropriate Si-to-CNT-to-C ratio also is helpful to the strength of the structure, but further analyses are needed for the correlation of structure design and mechanical properties.

The remarkable mechanical strength enables CNT@Si@C to undergo the calendering process, which is particularly important for Gr composite electrodes and practical application (The uncalendered electrodes have high porosity and it will lead to the increase of electrolyte amount and hence lower the cell energy density, even though the specific capacity/cycling stability of anode may be good. This has been largely neglected in most of previous battery research.). Supplementary Fig. 36 shows the top and cross-section views of electrodes with different calendering treatment. In spite of the flattened surface, the CNT@Si@C in the electrodes calendered to ~1.2 g cm^−3^ (20 µm thick) still preserves a spherical morphology, similar to that in as-casted electrodes.

### Electrochemical characterization of CNT@Si@C and graphite composite anodes

**Fig. 4:**
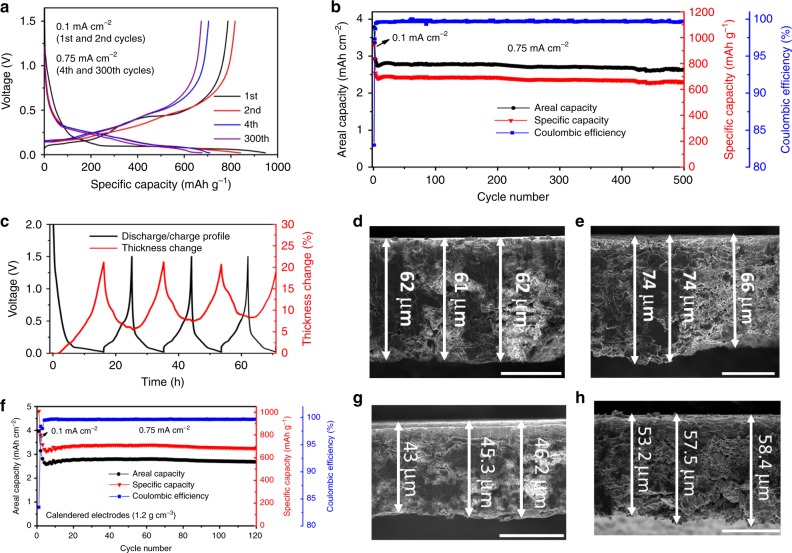
Electrochemical characterization of the CNT@Si@C-graphite anodes (30 wt% : 58 wt%) in half-cells. **a** Typical voltage curves of a CNT@Si@C and graphite composite anode with ~3 mAh cm^−2^ loading. The mass loading of the electrodes in Fig. 4 is 4.0 mg cm^−2^. **b** Cycling behavior of the same anode in **a**. **c** In-situ measurement of electrode swelling during discharge–charge at 0.2 mA cm^−2^. **d** SEM image of a pristine electrode (scale bar = 30 µm). **e** SEM image of a fully lithiated electrode after 1 cycle (scale bar = 30 µm). **f** Cycling stability of an electrode calendered to 1.2 g cm^−3^. **g** SEM image of an electrode after calendering to 1.2 g cm^−3^ while before cycling (scale bar = 30 µm). **h** SEM image of the calendered electrode at full lithiation state after 120 cycles (scale bar = 30 µm).

With the unique nanostructure of outstanding mechanical and electrochemical performance, the CNT@Si@C microspheres were mixed with Gr to prepare practical composite electrodes. Figure 4 shows the electrochemical performance of high loading CNT@Si@C-Gr composite electrode (30 wt% : 58 wt%) obtained in half-cells. The charge–discharge curves (Fig. 4a) showed typical profiles for the Si-Gr anodes between 0.02 and 1.5 V, similar to that previously reported in the literature^27,41,51^. The first cycle CE of the CNT@Si@C-Gr composite electrode is ~84%, higher than that of CNT@Si@C (75%; see Fig. 2 and Supplementary Fig. 21). The specific capacity is ~844 mAh g^−1^ (second formation cycle) at low current density of 0.1 mA cm^−2^ and ~718 mAh g^−1^ at 0.75 mA cm^−2^, indicating good rate performance even at a practical loading of ~3 mAh cm^−2^.

From the fact that the CNT@Si@C has good cycling stability (Fig. 2) and minimized particle swelling of ~40% (Fig. 2), the composite anodes of CNT@Si@C-Gr can be expected to have excellent cycling performance and minimized electrode swelling. Figure 4b shows excellent cycling life of CNT@Si@C-Gr with >92% capacity retention over 500 cycles. The CE at stable cycling is ~99.9%.

In-situ electrochemical dilatometer measurement and ex-situ SEM studies of the pristine electrode and the electrode at 100% SOC showed a consistent result of small electrode swelling (Fig. 4c–e). Figure 4c shows the swelling (red) of a typical high loading electrode upon cycling (black). The largest electrode expansion is ~21% at full lithiation and ~6% at delithiation states. Figure 4d–e are cross-section SEM images of a pristine electrode and an electrode after the first lithiation to 100% SOC. The electrode thickness before testing is ~50.7 µm in average [61.7 µm (the total thickness in average) − 11 µm (the thickness of Cu foil)], whereas the average electrode thickness at the first full lithiation is ~59.7 µm (70.7 µm − 11 µm). The electrode expansion at full lithiation is ~18.3% [(59.7 − 50.7)/50.7], consistent with the in-situ dilatometer result.

The extraordinary mechanical strength of the nanostructure CNT@Si@C enables the electrodes to be calendered to high densities. Supplementary Fig. 37 shows the calendered electrodes with the spherical morphology of CNT@Si@C maintained even when the electrodes were pressed to a density up to 1.4 g cm^−3^. The electrodes showed similar cycling stability with and without calendering. The electrode calendered to ~1.2 g cm^−3^ shows 96% capacity retention over 120 cycles (Fig. 4f), whereas the electrode calendered to ~1.4 g cm^−3^ has ~91% capacity retention (Supplementary Fig. 38). The specific capacities also are similar to the non-calendered electrodes even though the electrode loading is ~3 mAh cm^−2^. The polarization introduced by calendering is negligible in this case. The volumetric capacity densities of the electrodes of 1.2 and 1.4 g cm^−3^ are ~ 844 and 980 mAh cm^−3^ (Supplementary Table 2), calculated according to the second discharge capacities, ~799 and 796 mAh g^−1^, respectively (Fig. 4f and Supplementary Fig. 38). This is ~1.4 to 1.7 times of Gr electrodes^52^.

The SOC and EOL swelling of the calendered electrodes can be estimated from SEM analyses of the electrode thickness change before and after cycling. For the electrode calendered to 1.2 g cm^−3^ density, the swelling at 100% SOC after the first cycle is ~23.5% [(42.6 − 34.5)/34.5] (Supplementary Fig. 39). In the case of EOL swelling, the thickness increased from 33.8 µm (44.8 (the average thickness of the entire electrode) − 11 (the Cu foil thickness), Fig. 4g and Supplementary Table 2) to ~45.4 µm (56.4 − 11, Fig. 4h and Supplementary Table 2) after 120 cycles. The EOL swelling is ~34% [(45.4 − 33.8)/33.8]. The electrode calendered to 1.4 g cm^−3^ shows thickness increase from ~28.1 µm (Supplementary Fig. 26 and Supplementary Table 2) to ~40.7 µm (Supplementary Fig. 38) after 120 cycles. The EOL swelling is ~45%.

**Fig. 5:**
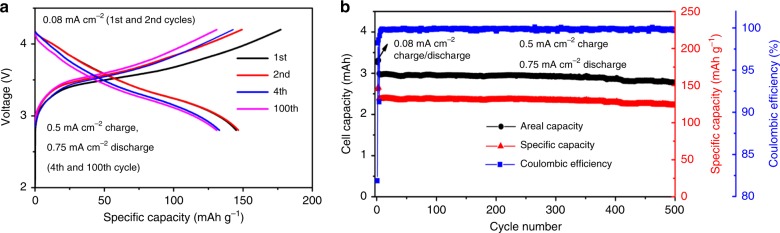
Electrochemical performance of a typical full cell with Li(Ni_1/3_Mn_1/3_Co_1/3_)O_2_ cathode and pre-cycled CNT@Si@C and graphite composite anode. **a** Voltage profiles. **b** Long-term cycling data of the full cell in **a**.

CNT@Si@C and Gr composite anodes also show excellent performance in full cells against Li (Ni_1/3_Mn_1/3_Co_1/3_)O_2_ (NMC) cathodes. NMC cathodes were prepared with appropriate loading (see experiment part for details) and tested at different current densities in a half-cell configuration. With an areal loading of ∼2.4 mAh cm^−2^, the NMC cathode delivers a specific capacity of ∼150 mA h g^−1^ between 2.7 to 4.3 V (Supplementary Fig. 40). The typical full cell with the pristine CNT@Si@C-Gr anode has ~77% first cycle CE and 76% capacity retention over 120 cycles (Supplementary Fig. 41). In order to further improve the cycling performance of full cells, the anodes were first cycled in a half-cell configuration against Li metal before the full-cell test (see Methods part for details). Good full-cell performance was obtained with an *n*/*p* ratio of ~1.1:1 (anode/cathode mass ratio = 0.22). Figure 5 presents a typical full-cell performance with NMC333 cathode and pre-cycled CNT@Si@C-Gr anode of ~3 mA h. Figure 5a shows the charge/discharge voltage curves of a typical full cell cycled between 2.8 and 4.2 V. The specific discharge capacity of NMC cathode is ~145 mA h g^−1^_,_ similar to those from the half-cell measurement. It also demonstrates excellent cycle life with capacity of 92% in 500 cycles (Fig. 5b).

## Discussion

On the basis of its excellent electrochemical performance, mechanical strength, and structural integrity demonstrated above, CNT@Si-based hierarchical porous nanostructure holds great promise for practical next-generation battery applications. It not only can fulfill the performance requirements of practical Si-based anodes in cycling stability, CE, high volumetric capacity density, minimized electrode initial, and EOL swelling, but is also suitable for standard industrial processing procedures including calendering. The designed CNT@Si@C is among the top performance Si anodes (Supplementary Table 3) and provides insight for design Si anodes towards practical applications. Further development of CNT@Si-based hierarchical porous nanostructure using other scalable and economical methods, such as spray-drying or mechanical condensation will enable next generation of high-energy Li-ion batteries. The rational design of nanostructured battery materials and electrodes in this work also opens a new dimension in material design for other batteries.

## Methods

### Material synthesis

The hierarchical porous CNT@Si microspheres were prepared by aluminothermic reduction of the CNT@SiO_2_ microspheres from sol-gel and emulsion synthesis (see details in Supplementary Materials). Briefly, CNT (cheap Tubes, outer diameter: ~30–50 nm, insider diameter: ~5–10 nm) was coated with polyvinylpyrrolidone (PVP, Sigma-Aldrich, M.W. 40,000) by 2 h sonication in a 0.5 wt% PVP water solution. The surface modified CNT after filtration was dispersed in an ethanol and H_2_O solution (10 : 1 volume ratio). Appropriate amount of NH_3_·H_2_O (25 wt%) and tetraethoxysilane (TEOS) were added into the above dispersion slowly. The product was filtered after 24 h and washed with ethanol and deionized water, and re-dispersed in water. The CNT@SiO_2_ water dispersion was mixed with 1-octadecene solution (0.3 wt%) in a 1 : 8 volume ratio and homogenized to form emulsion. An aqueous suspension of the silica-coated carbon tubes (CNT@SiO_2_) forms numerous micrometer-sized water droplets dispersed in the oil phase. The mixture was heated at 95~98 °C for 4 h. During the heating process and water evaporation, colloidal particles aggregate and the CNT@SiO_2_ cables in the water droplets condensed to form CNT@SiO_2_ microspheres. The mixture then was centrifuged to collect CNT@SiO_2_ microspheres, which later were washed with petroleum ether. The final powder was annealed at 550 °C for 1 h in argon for removal of the organic molecules and condensation of the CNT@SiO_2_ microspheres. Then, a mixture of CNT@SiO_2_, AlCl_3_ and Al metal (weight ratio of ~1 : 6 : 1.6) was sealed in a Swagelok® reactor and heated for 15 h at 350 °C under Ar atmosphere. After cooling, the products were first immersed in H_2_O and subsequently in 1 M HCl to remove byproducts. The product of CNT@Si was collected after filtration, washing with H_2_O and ethanol, and vacuum-drying. CVD carbon coating of CNT@Si was conducted at 700 °C for 30 min using acetylene as the carbon source^28^.

### Characterization

Surface morphology and cross-section of the microspheres and electrodes were studied using a focused ion-beam scanning electron microscope (FEI Helios Nanolab) with EDS features. For the measurement of initial and EOL swell, the cells were disassembled with anodes at 100% lithiated state. Before SEM measurement, the electrodes were washed with anhydrous dimethyl carbonate for three times and dried completely in a glovebox’s antechamber under vacuum. TEM, HRTEM, and EDS elemental line scan and mapping were carried out on FEI Titan 80–300 STEM equipped with a condenser lens Cs corrector. In-situ swelling of CNT@Si and CNT@Si@C microspheres upon lithiation was investigated by a solid-state “nano-battery” configuration^28^. The porous structure of the samples were determined by nitrogen adsorption measurement using a Micromeritics ASAP 2020.

### Mechanical property measurement and simulation

Mechanical strength of the CNT@Si@C microspheres was investigated by in-situ AFM–SEM test. An AFM probe with a spring constant of 40 N m^−1^ was attached to the sample holder with the conducting tape. The AFM tip was fabricated to cylindrical shape from triangular pyramid that was pre-oriented, cut, and polished by focused ion-beam milling. The diameter of the tip are designed within an appropriate range of ~1.5–3 µm. A random picked Si@CNT@C microsphere was attached to the tungsten probe with Pt glue. The sphere was bought into contact with the prepared AFM tip by the 3D-piezo-manipulator and pressure was applied to the particle by exerting a displacement against the AFM tip. The force acting on the particle was calculated by measurement of the displacement of AFM tip during the pressing. The FE software Abaqus CAE 6.14-4 (SIMULIA, 2015) was used to simulate the particle deformation. The symmetries of the modeled specimens were considered to lower the computation time. In the simulations, four-node axisymmetric element (CAX4R) with total 6000 elements was used. The sample was deformed by a same displacement in the experiment.

### Battery tests and electrode swelling study

The anodes were prepared by mixing CNT@Si@C material, Super P, and dreambond® polyimide (PI) binder in a weight ratio of 80 : 5 : 15. The CNT@Si@C-Gr composite electrodes were composed of 30 wt% CNT@Si@C, 58 wt% Gr, 2 wt% Super p, and 10 wt% polyacrylic acid (PAA) binder. PAA binder is used for the CNT@Si@C and Gr composite electrode, because PI binder does not work well for the electrode with large amount of Gr, which has large particle size of tens of micrometers. The cathodes were prepared by mixing 96 wt% NMC, 2 wt% Super P, and 2 wt% polyvinylidene difluoride binder (2.5 wt% in *N*-methyl-2-pyrrolidone). The solution (1 M) of LiPF_6_ in ethylene carbonate/diethyl carbonate (3 : 7, w/w) and 10 wt% fluoroethylene carbonate was used as the electrolyte for all the battery tests. The electrochemical performance was evaluated using galvanostatic charge–discharge protocols. For half-cells, Si-based electrodes were cycled between 0.02 V and 1.5 V (vs. Li/Li^+^), whereas the NMC cathodes were tested from 2.7 V to 4.3 V. The specific capacity was calculated based on the total weight of the active composite material such as CNT@Si@C, CNT@Si@C-Gr, or NMC, etc. Electrode thickness change during lithiation/delithiation was monitored by an electrochemical dilatometer (EL-Cell, Germany). For the full cells, the CNT@Si@C-Gr electrodes were first pre-cycled in half-cell configuration for three cycles at 0.08 mA cm^−2^. The voltage window was 0.02–1.5 V and all the cells were collected when they were de-lithiated to 1.5 V. After three formation cycles in half-cells, a stable SEI was expected to form on anodes. The full cells were tested between 2.8 and 4.2 V. The full-cell-specific capacity was calculated based on cathode.

## Supplementary information

Supplementary Information

Description of Additional Supplementary Files

Supplementary Movie 1

Supplementary Movie 2

Supplementary Movie 3

Supplementary Movie 4

Supplementary Movie 5

Supplementary Movie 6

Supplementary Movie 7

## Data Availability

The data that support the findings of this study are available from the corresponding authors upon reasonable request.
