# Supplementary material for: Hierarchical porous silicon structures with extraordinary mechanical strength as high-performance lithium-ion battery anodes
Source: Nat Commun. 2020 Mar 19;11:1474. doi: 10.1038/s41467-020-15217-9 (PMC7081208; doi:10.1038/s41467-020-15217-9)
Supplement: Supplementary file 1 — Supplementary Information [file 41467_2020_15217_MOESM1_ESM.pdf]

Supplementary Information

Hierarchical Porous Silicon Structures with Extraordinary Mechanical Strength as High-Performance Lithium-Ion Battery Anodes

Haiping Jia et al

Supplementary Figures

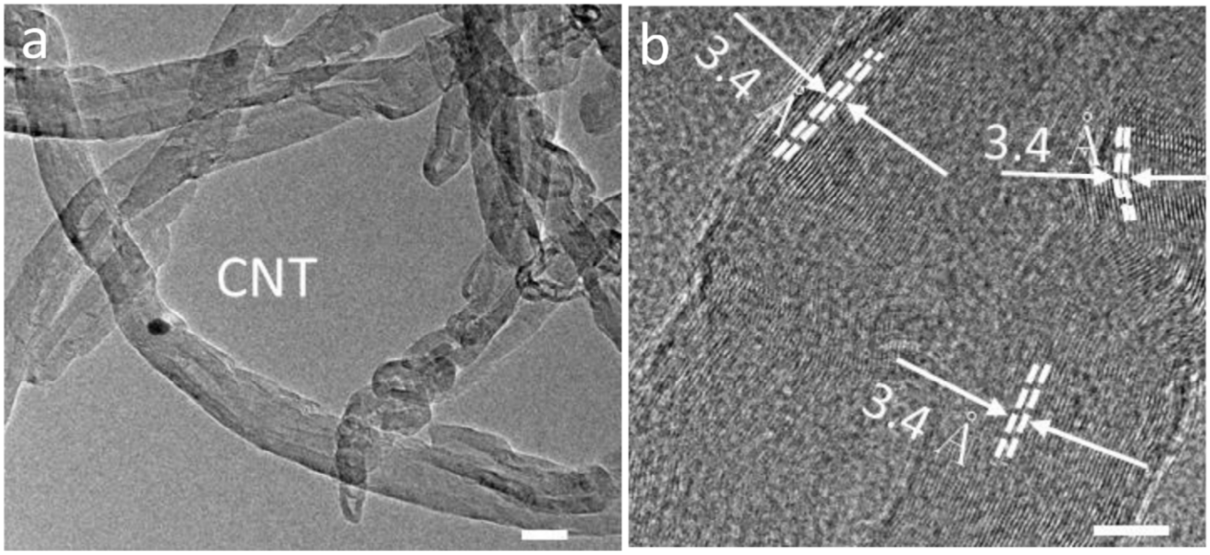

Supplementary Figure 1. Typical TEM images of carbon nanotubes. (a) Low magnification TEM image (scale bar = 20 nm). **(b)** High resolution TEM image showing the lattice fringes of the CNT wall of ~3.4Å (scale bar = 5nm).

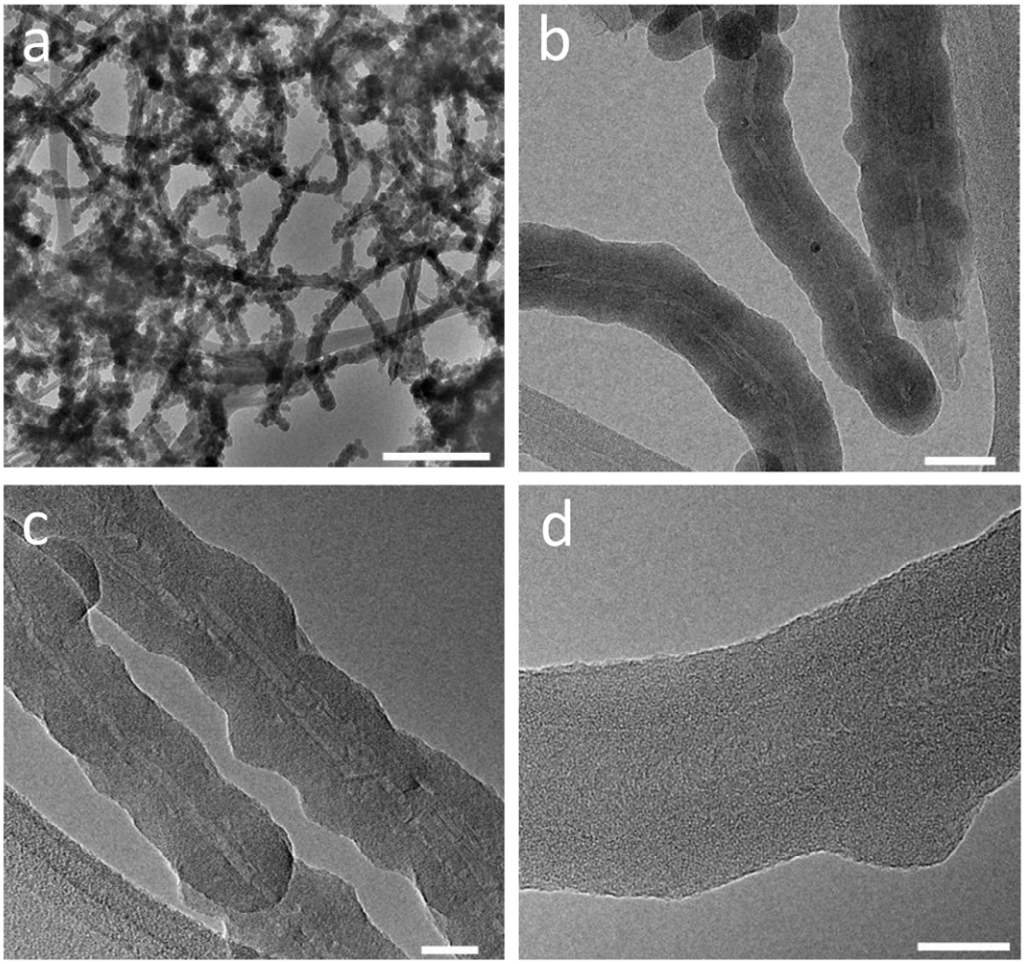

Supplementary Figure 2. **Morphology characterization of CNT@SiO_2 coaxial cables. (a)**TEM image (scale bar = 500 nm) and **(b-d)** High-resolution transmission electron microscopy (HR-TEM) images (scale bar for b, c and d =50, 20 and 20nm, respectively) of CNT@SiO_2 coaxial cables.

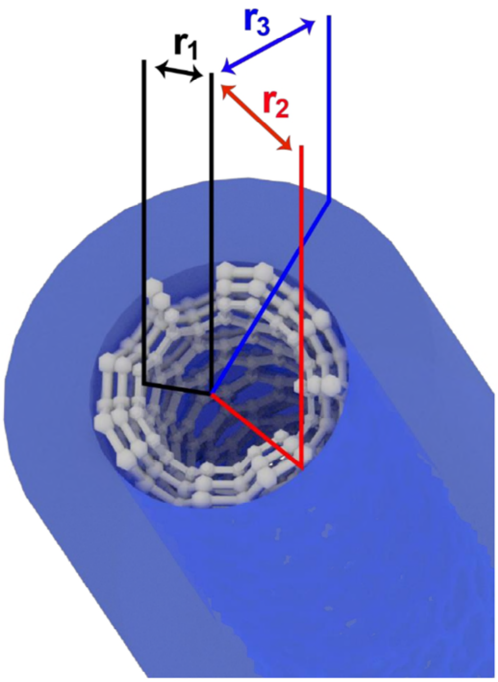

Supplementary Figure 3. Schematic of the design of the CNT@SiO_2 core-shell coaxial cables.

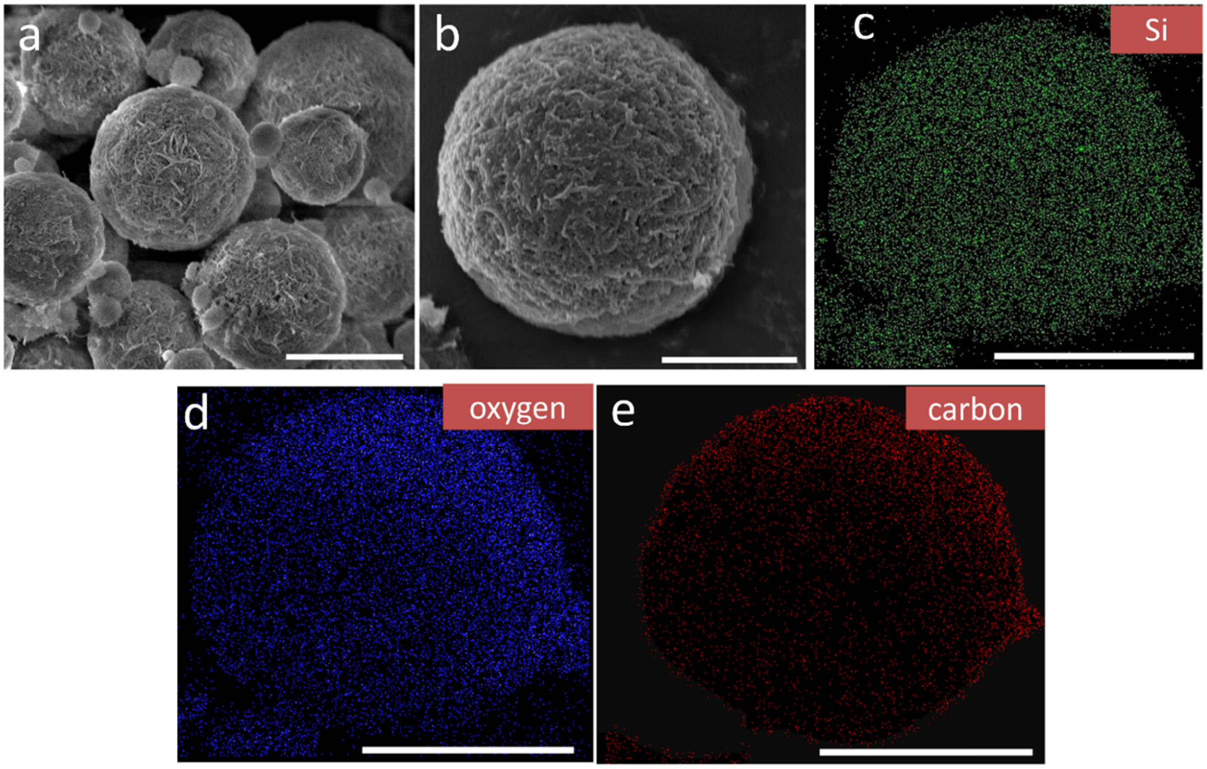

Supplementary Figure 4. SEM images and the corresponding EDS mappings of CNT@SiO_2 microspheres. (a) Low magnification SEM image (scale bar = 4 µm); **(b)** Zoom-in SEM image (scale bar = 3 µm); **(c)** Si map (scale bar = 3 µm); **(d)** Oxygen map (scale bar = 3 µm); **(e)** carbon map (scale bar = 3 µm).

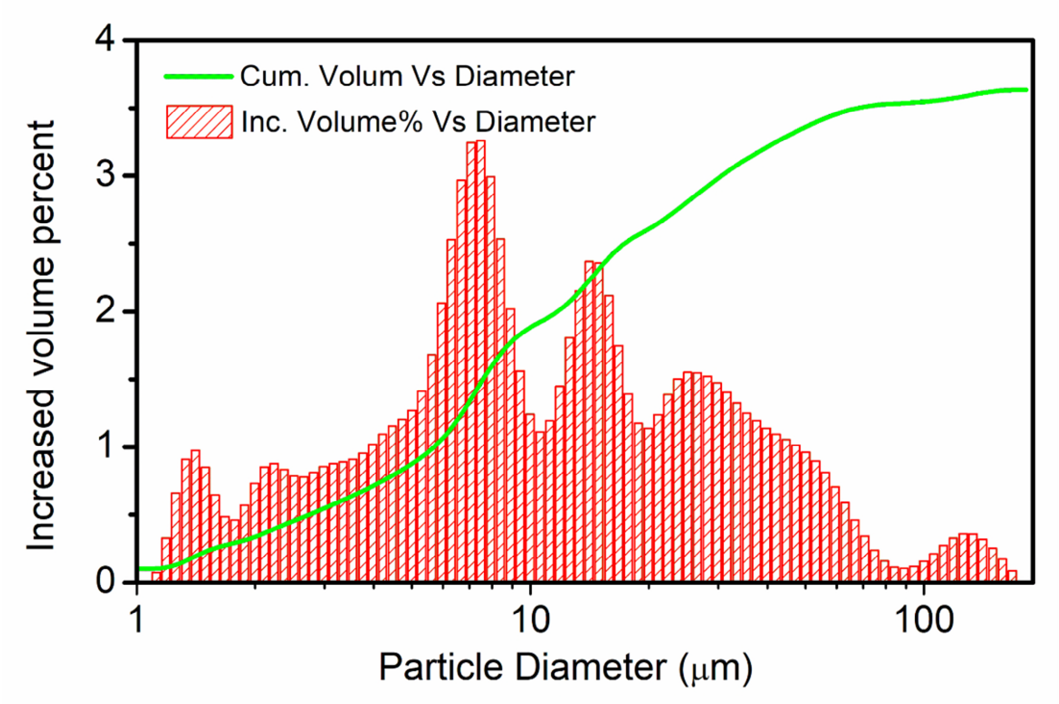

Supplementary Figure 5. Particle size distribution of CNT@SiO_2 microspheres.

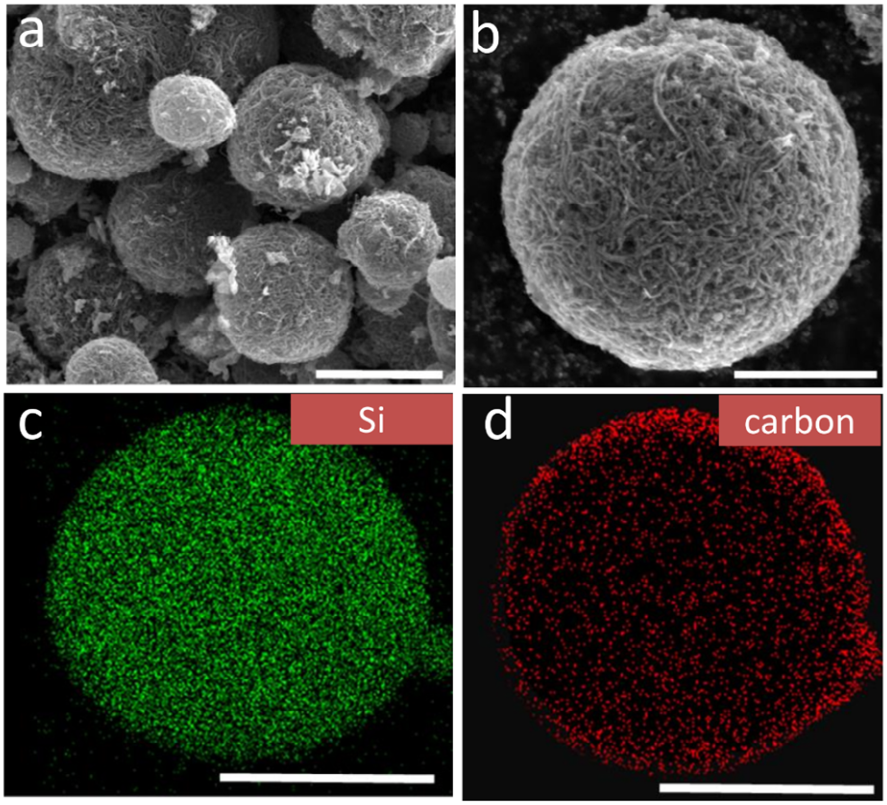

Supplementary Figure 6. **SEM images and EDS mappings of CNT@Si microspheres. (a)** low magnification SEM image (scale bar = 5 μm); (b) zoom-in SEM image (scale bar = 4 μm); (c) Si map (scale bar = 4 μm); (d) carbon map (scale bar = 4μm).

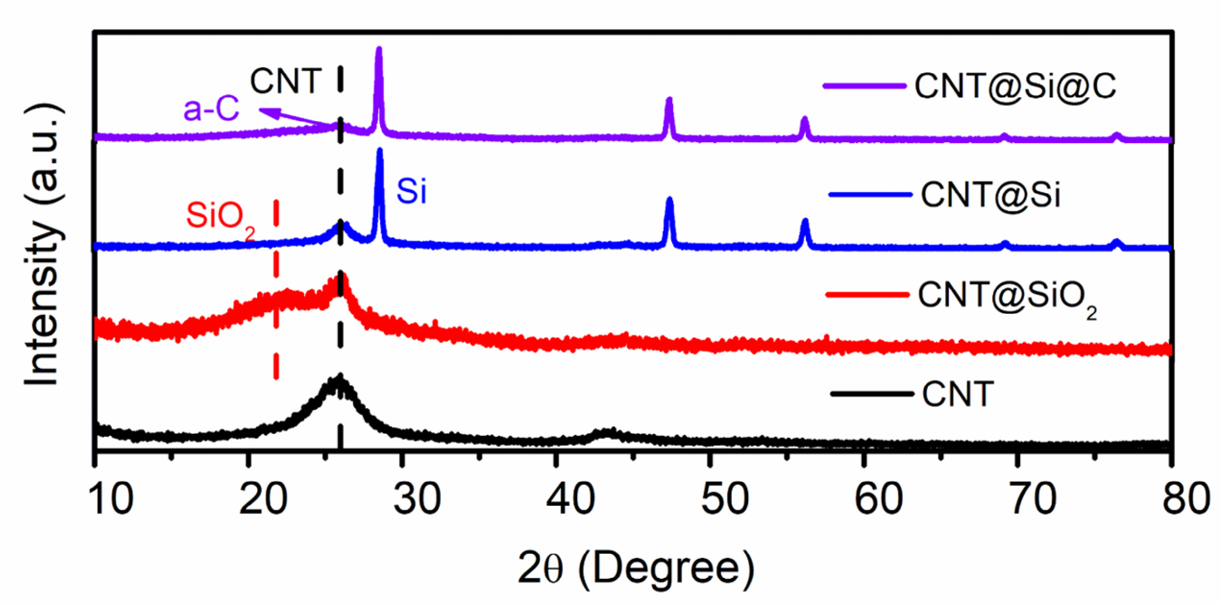

Supplementary Figure 7. XRD patterns of CNT, CNT@SiO_2, CNT@Si and CNT@Si@C.

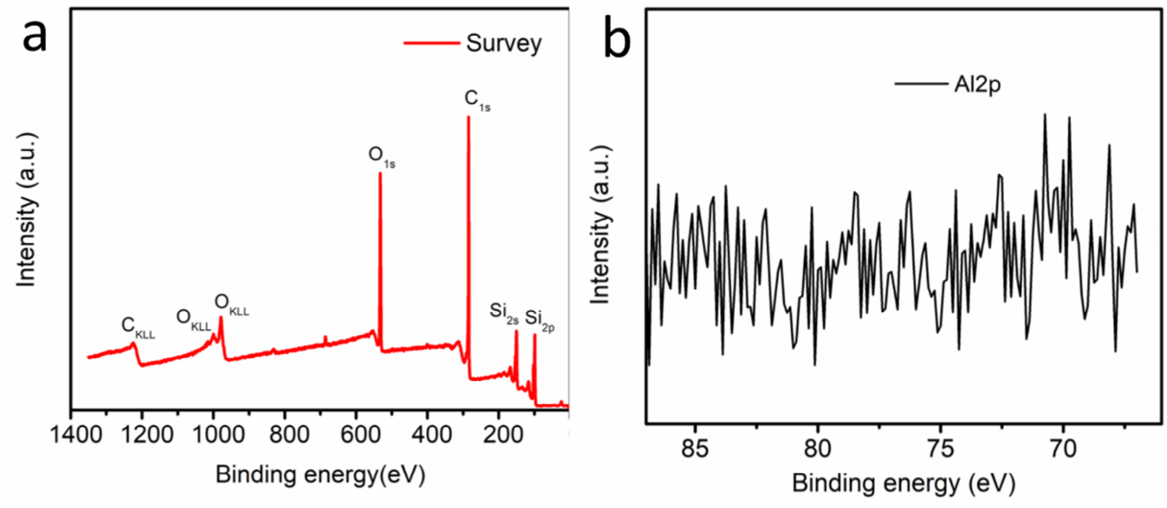

Supplementary Figure 8. **XPS spectra characterization for CNT@Si. (a)** XPS wide scan and **(b)** high-solution spectrum Al2p of CNT@Si.

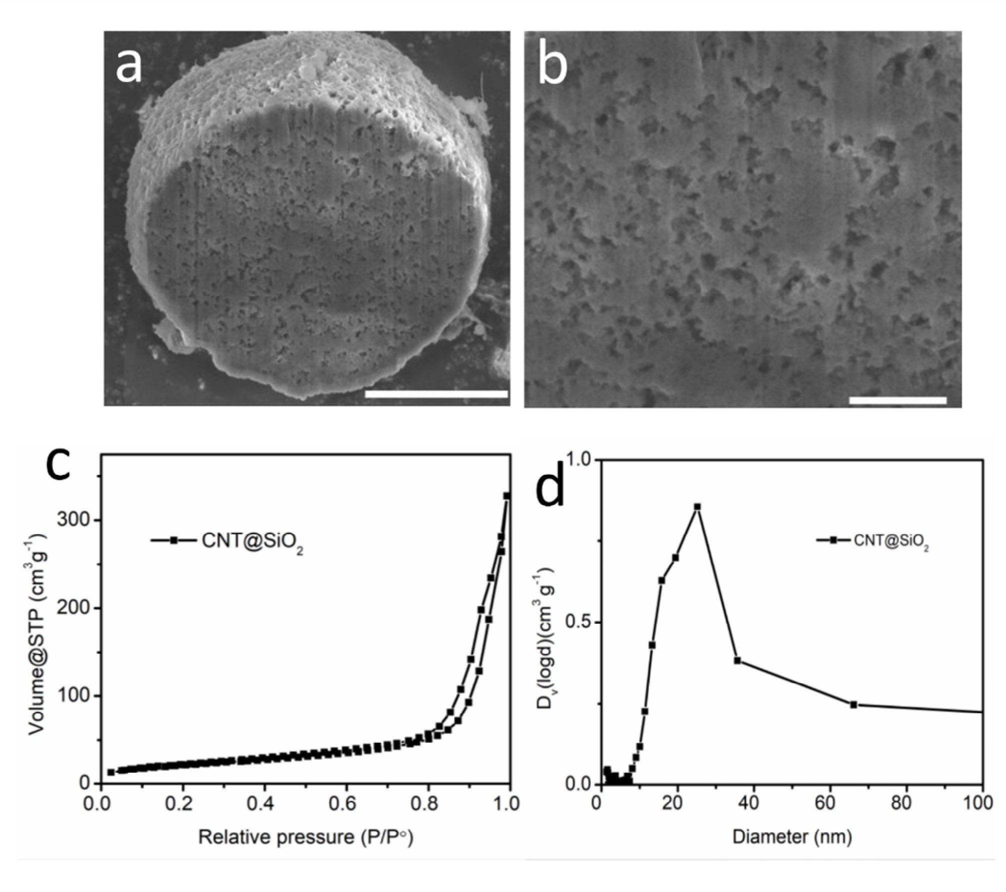

Supplementary Figure 9. **Characterization of the porous structure of $CNT@SiO_2$. (a, b)** Cross-section SEM images of a $CNT@SiO_2$ microsphere (scale bar for a and b = 4 µm and 1µm, respectively). **(c, d)** Isothermal curve and pore size distribution of $CNT@SiO_2$.

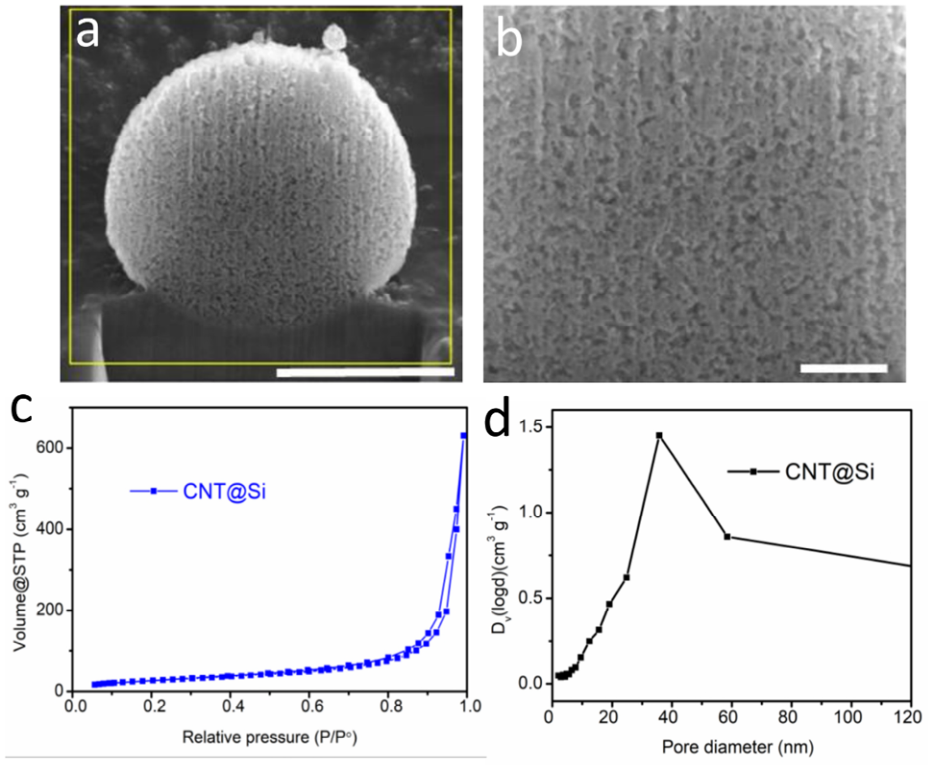

Supplementary Figure 10. **Characterization of the porous structure of CNT@Si. (a, b)** Cross-section SEM images of a CNT@Si particle (scale bar for a and b = 2 and 1 μm, respectively). **(c, d)** Isothermal curve and pore size distribution of CNT@Si.

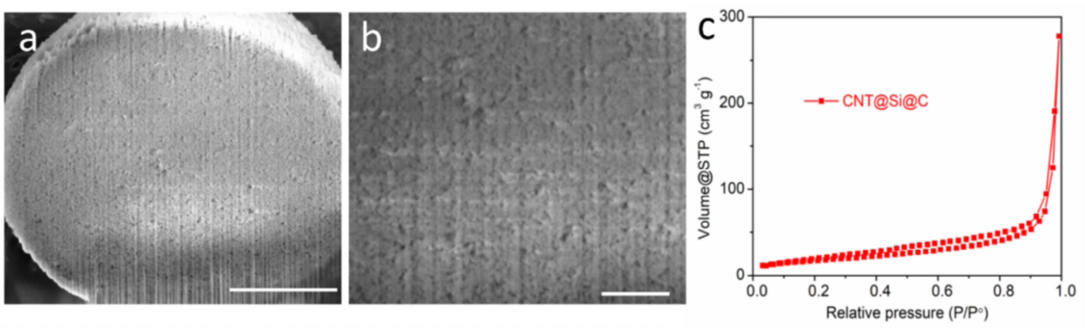

Supplementary Figure 11. **Characterization of the porous structure of CNT@Si@C. (a, b)** Cross-section of SEM images of a CNT@Si@C (scale bar for a and b = 4 μm and 1 μm). **(c)** Isothermal curve of CNT@Si@C.

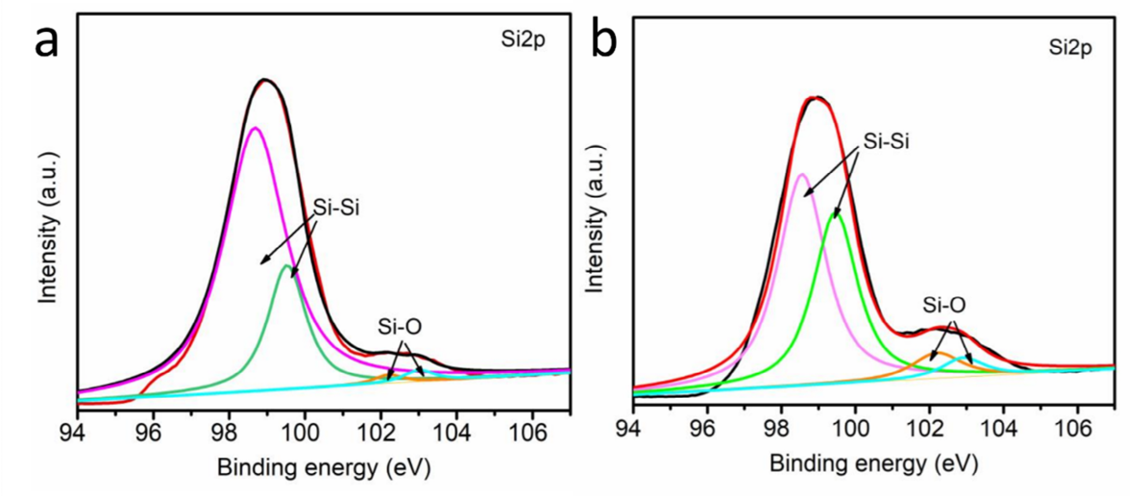

Supplementary Figure 12. XPS spectra characterization of CNT@Si and CNT@Si@C.

High-resolution XPS spectra of Si2p of **(a)** CNT@Si and **(b)** CNT@Si@C.

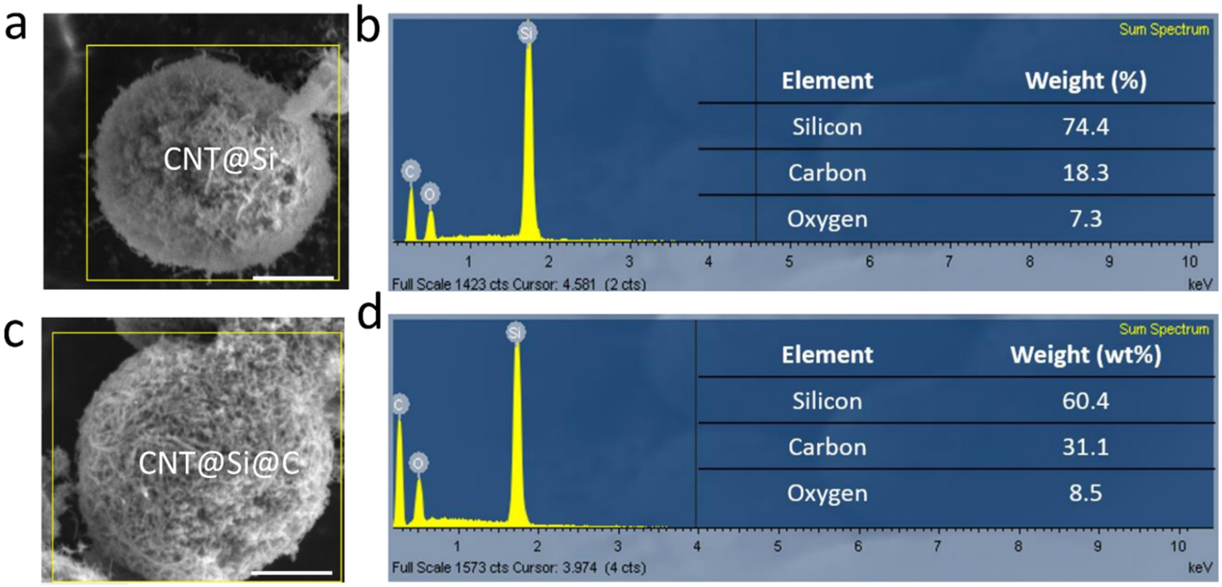

Supplementary Figure 13. Morphology characterization and composition analysis of CNT@Si and CNT@Si@C. (a, c) SEM image (scale bar = 1nm) and (b, d) EDS spectrum of CNT@Si and CNT@Si@C, with inset showing the detailed contents of different elements.

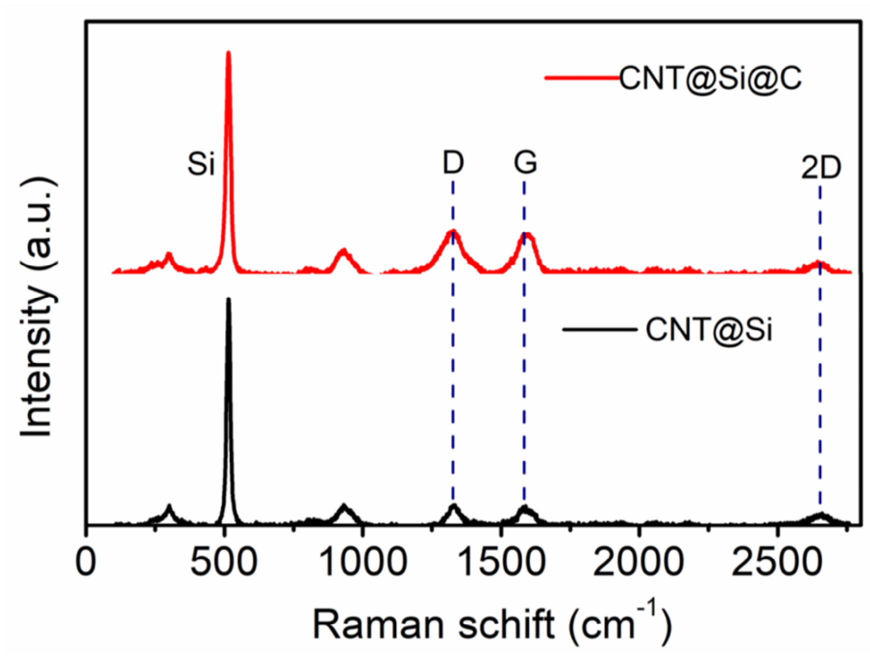

Supplementary Figure 14. Raman spectra of CNT@Si and CNT@Si@C.

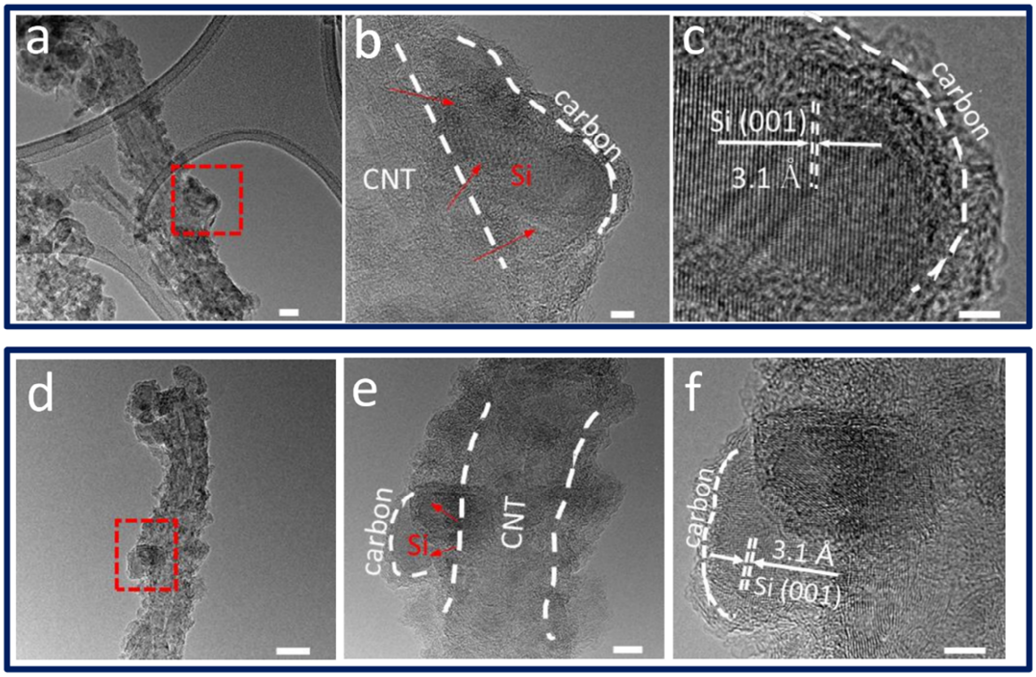

Supplementary Figure 15. **Morphology characterization of CNT@Si@C. (a-f)** HRTEM images of CNT@Si@C at different magnifications (scale bar for a, b, c, d, e and f = 20, 5, 5, 20, 5 and 5 nm, respectively).

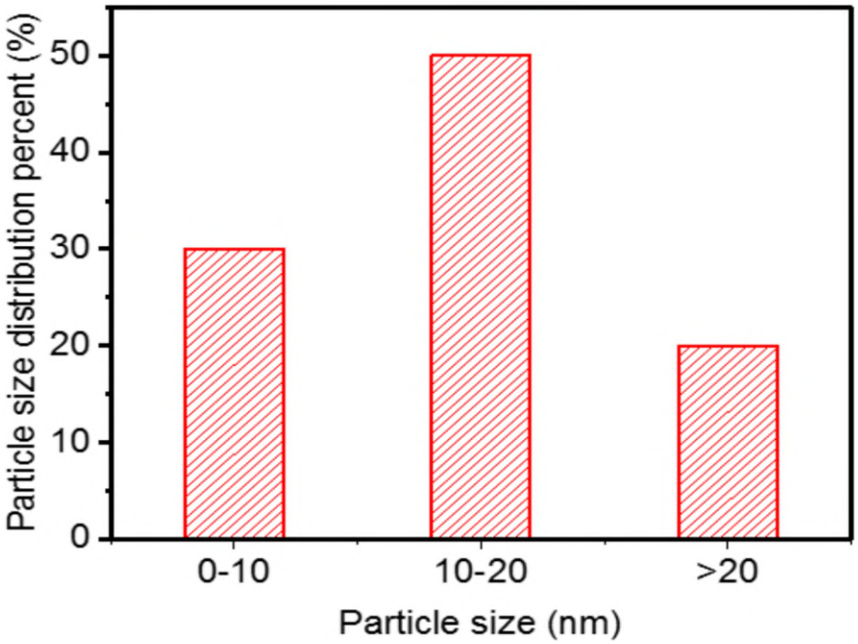

Supplementary Figure 16. Particle size distribution of silicon of CNT@Si@C microspheres.

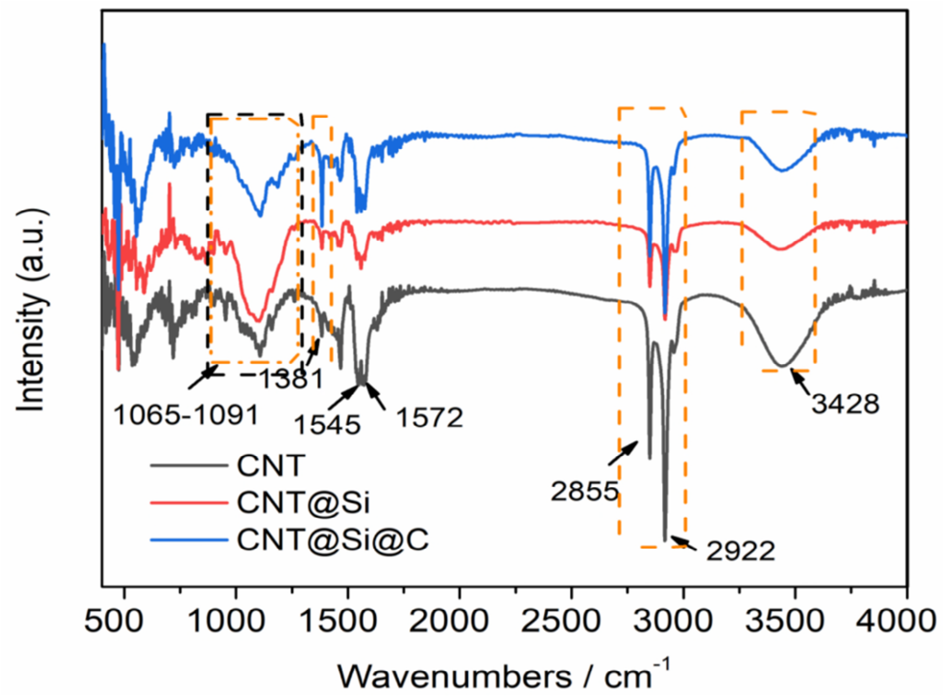

Supplementary Figure 17. FTIR spectra of CNT, CNT@Si and CNT@Si@C.

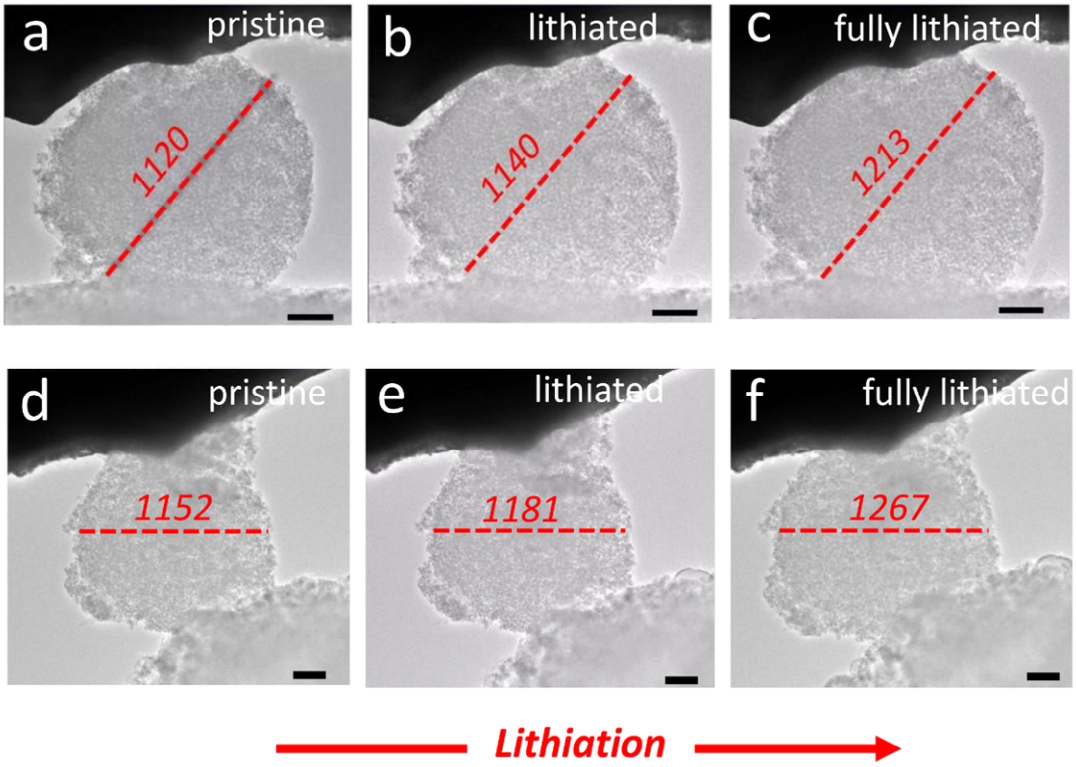

Supplementary Figure 18. **In-situ TEM observation of the lithiation process of two CNT@Si microsphere particles.** TEM images of the CNT@Si microsphere particles at the beginning **(a, d)** (scale bar = 0.2 μm), in the middle **(b, e)** (scale bar = 0.2 μm), and at the end of lithiation **(c, f)** (scale bar = 0.2 μm). The unit for the diameters in the figures is nm.

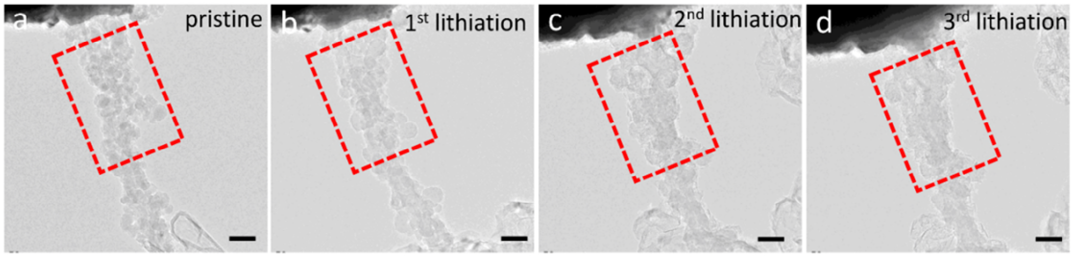

Supplementary Figure 19. **In-situ TEM characterization of CNT@Si@C cable.** TEM images of **(a)** the pristine, **(b)** after first lithiation, **(c)** after second lithiation and **(d)** after third lithiation (d). Scale bar = 50 nm.

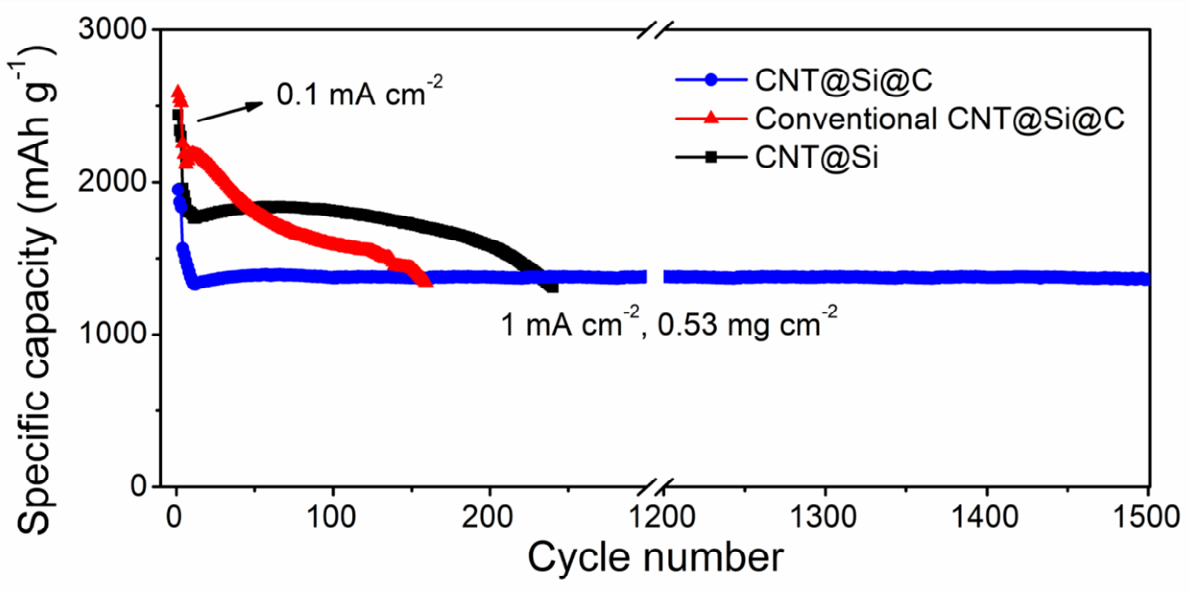

Supplementary Figure 20. Comparison of the long-term cycling performance of CNT@Si@C, CNT@Si microsphere and conventional CNT@Si@C nanostructure anodes.

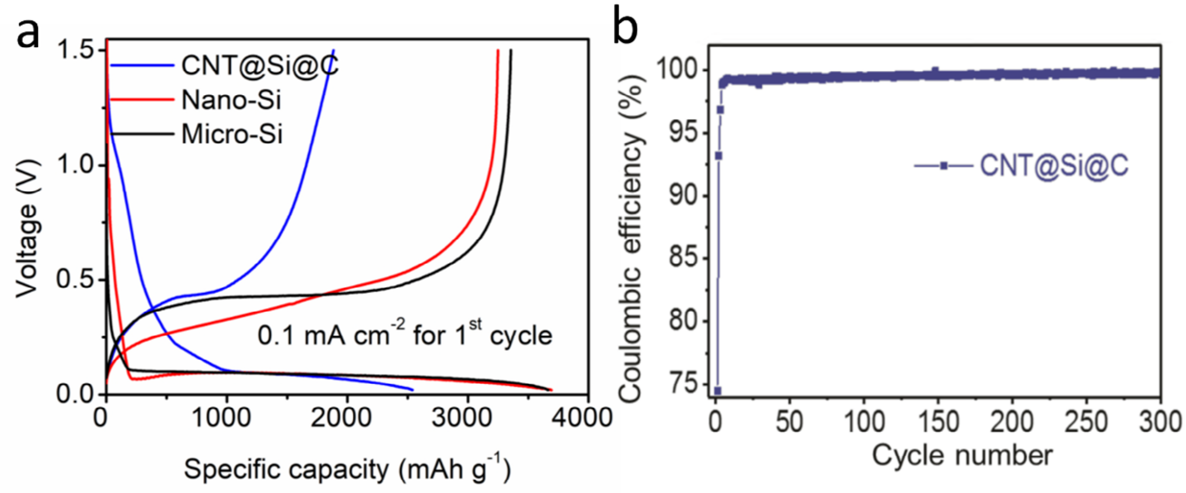

Supplementary Figure 21. Electrochemical performance of CNT@Si@C, nano-Si and micro-Si. (a) Voltage profiles of CNT@Si@C, nano-Si and micro-Si at the 1st cycle. **(b)** Coulombic efficiency of the CNT@Si@C electrodes at the mass loading of 0.53 mg cm^{-2}.

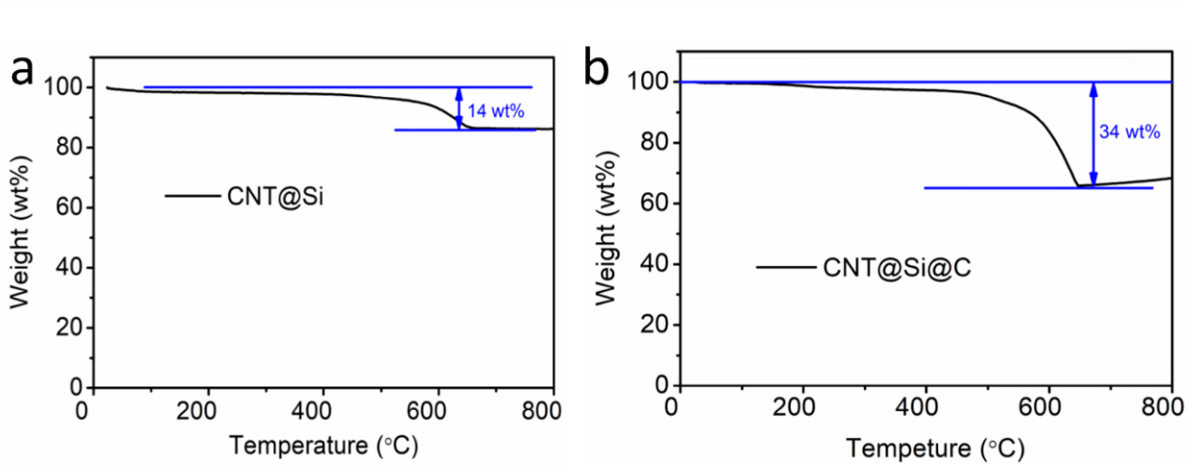

Supplementary Figure 22. Thermogravimetric analysis of **(a)** CNT@Si and **(b)** CNT@Si@C.

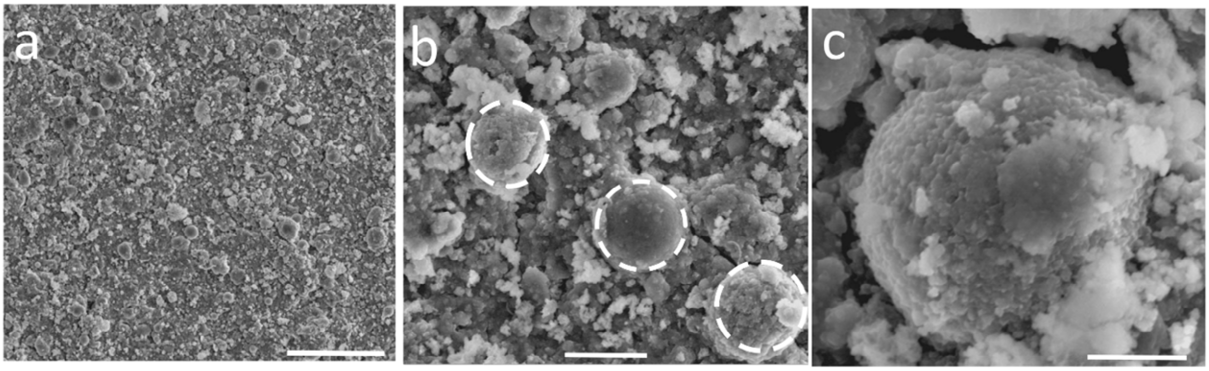

Supplementary Figure 23. SEM images of the CNT@Si@C electrodes after 500 cycles. (a-c) SEM images of CNT@Si@C electrodes at different magnifications (scale bar of a, b and c = 30, 5 and 2 μm, respectively).

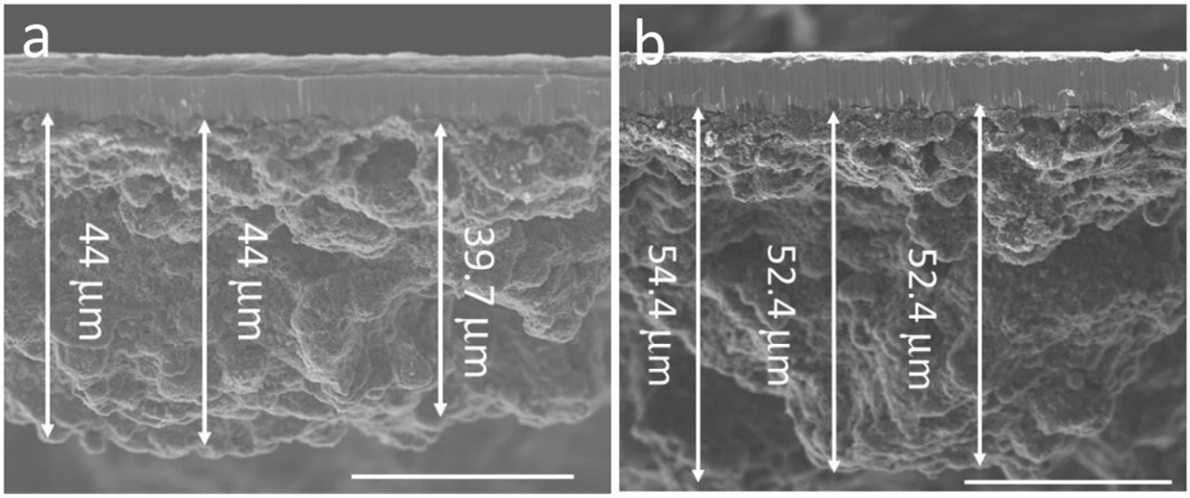

Supplementary Figure 24. Ex-situ SEM images of the CNT@Si@C electrodes (a) before cycling (scale bar = 30 µm) and **(b)** after initial lithiation (scale bar = 30 µm).

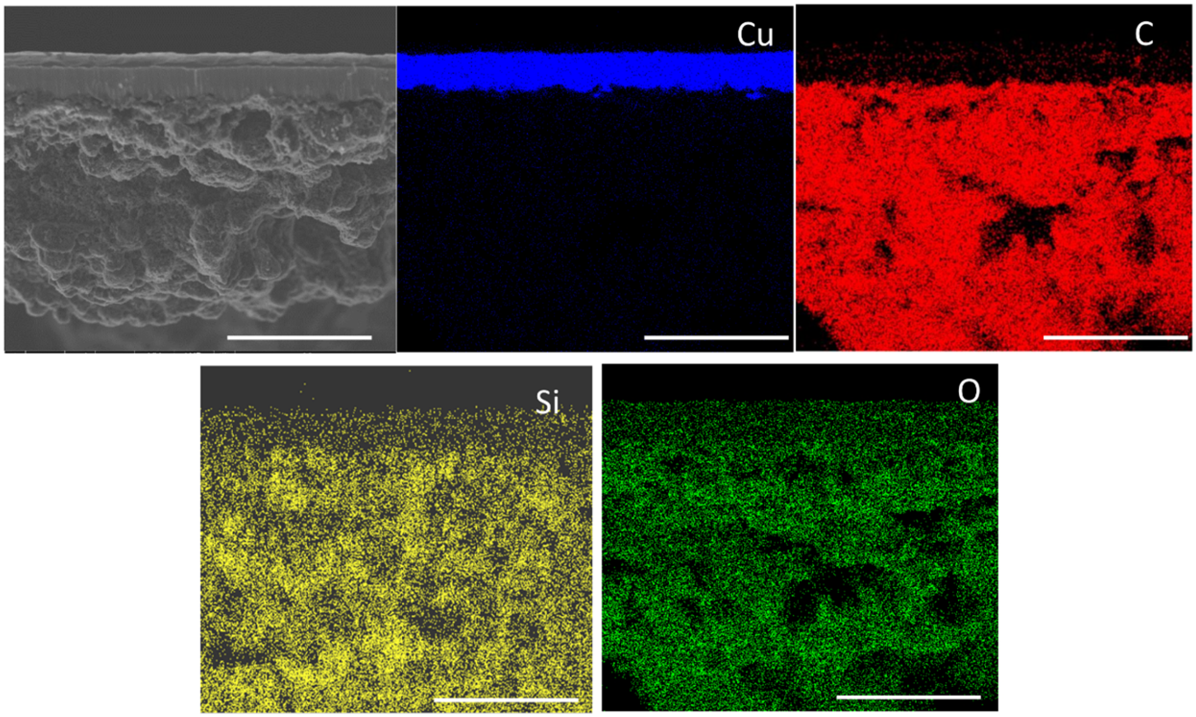

Supplementary Figure 25. EDS mapping from the pristine CNT@Si@C electrode before cycling (scale bar = 30 μm).

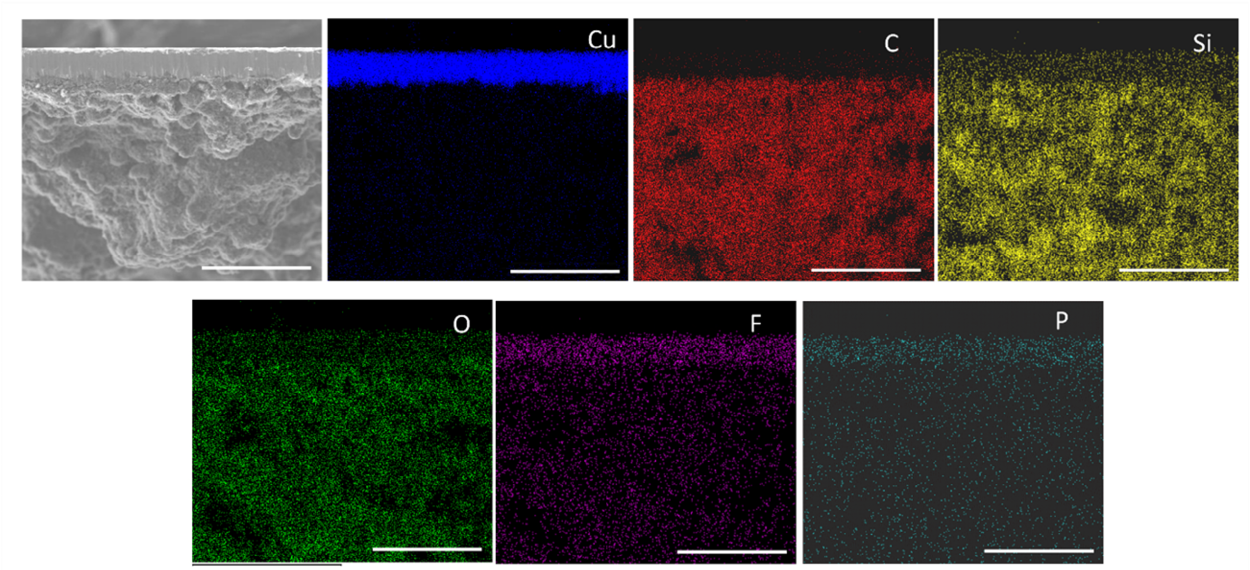

Supplementary Figure 26. EDS mapping from the CNT@Si@C electrode after initial lithiation (scale bar = 30 μm).

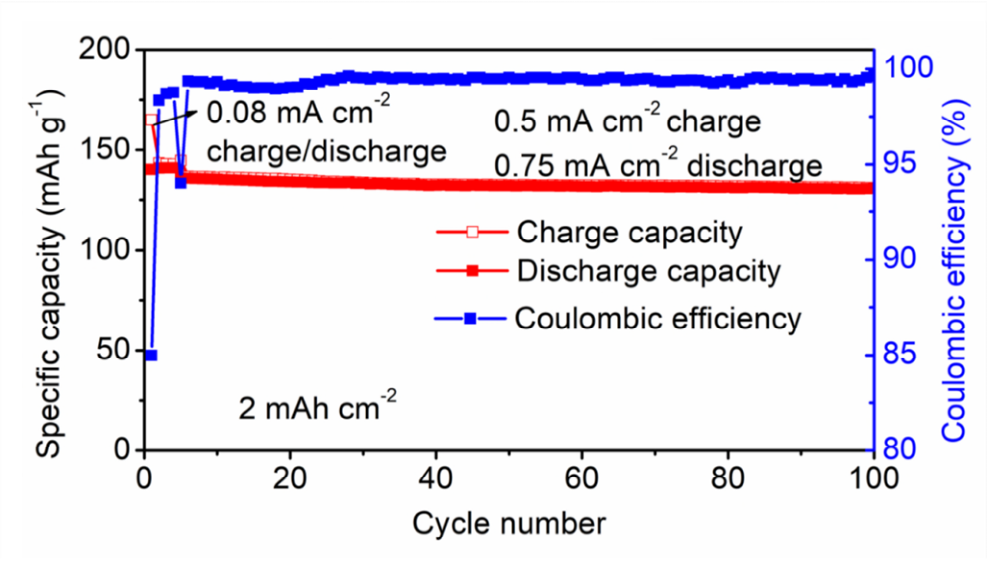

Supplementary Figure 27. Long-term cycling performance of CNT@Si@C||NMC333 full-cells.

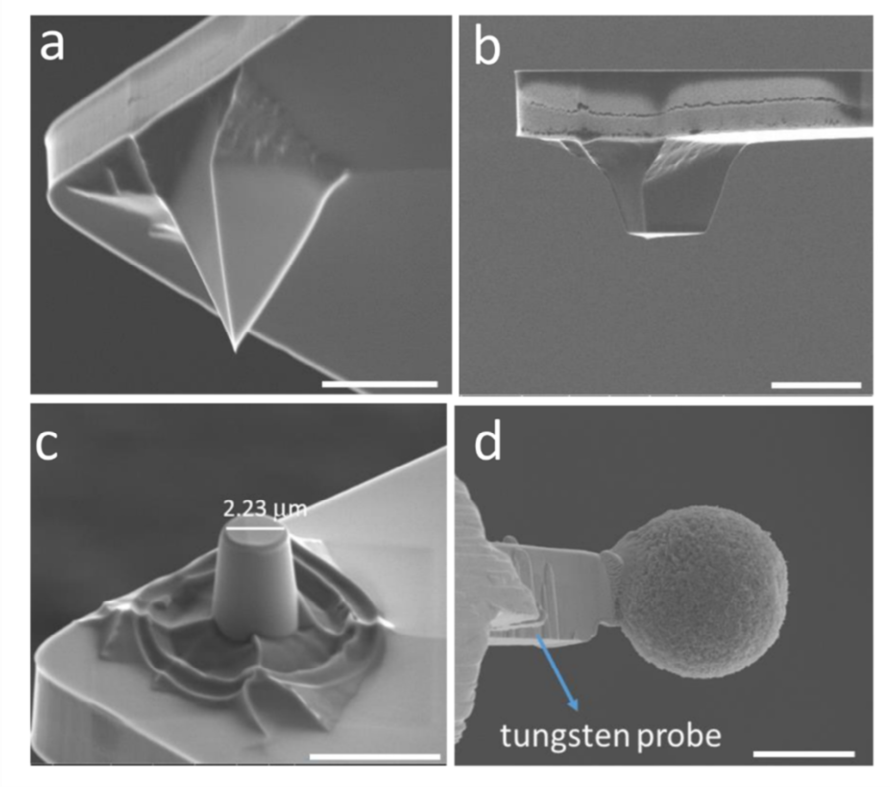

Supplementary Figure 28. SEM images showing the preparation of the AFM tip for in-situ AFM-SEM experiment. **(a-c)** AFM tip (scale bar = 5 µm) and **(d)** a CNT@Si@C microsphere particle attached to the tungsten probe of the 3D piezo-manipulator (scale bar = 5 µm).

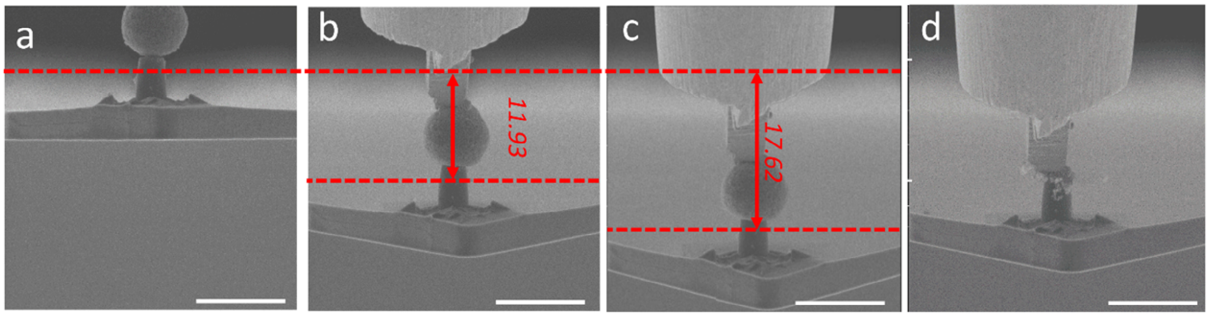

Supplementary Figure 29. Screenshots from the in-situ AFM-SEM experiment showing the AFM tip displacements/cantilever deflection from the beginning to the particle cracks (scale bar = 10 μm). The unit of the numbers in the figures is μm.

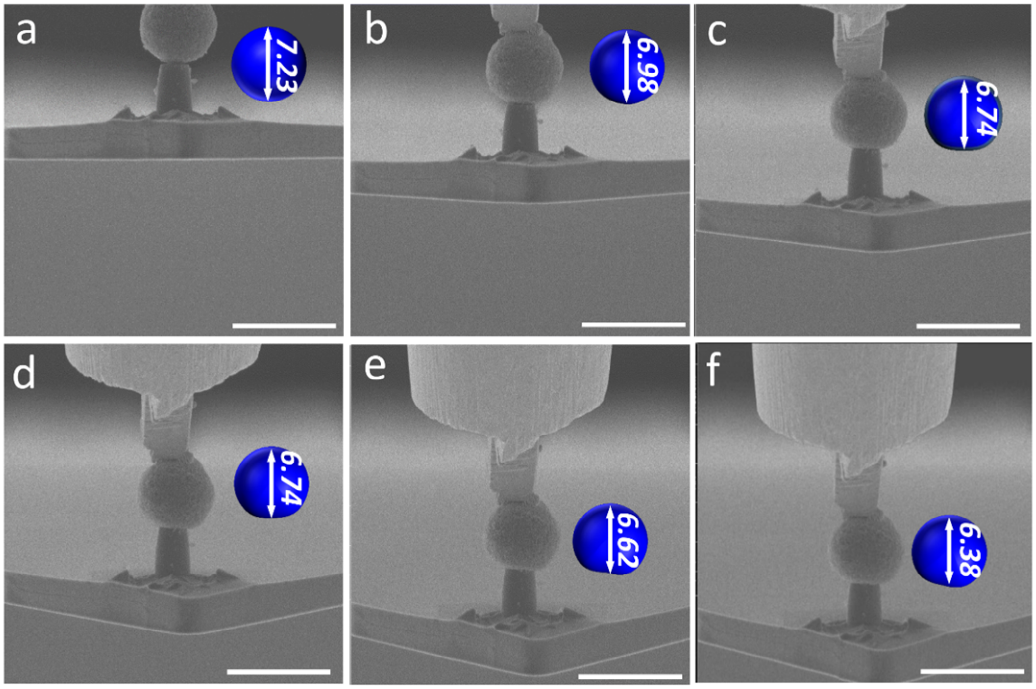

Supplementary Figure 30. Screenshots from the AFM-SEM experiment showing the deformation of the CNT@Si@C particle under an applied force (scale bar = 10 μm). The unit of the numbers in the figures is μm.

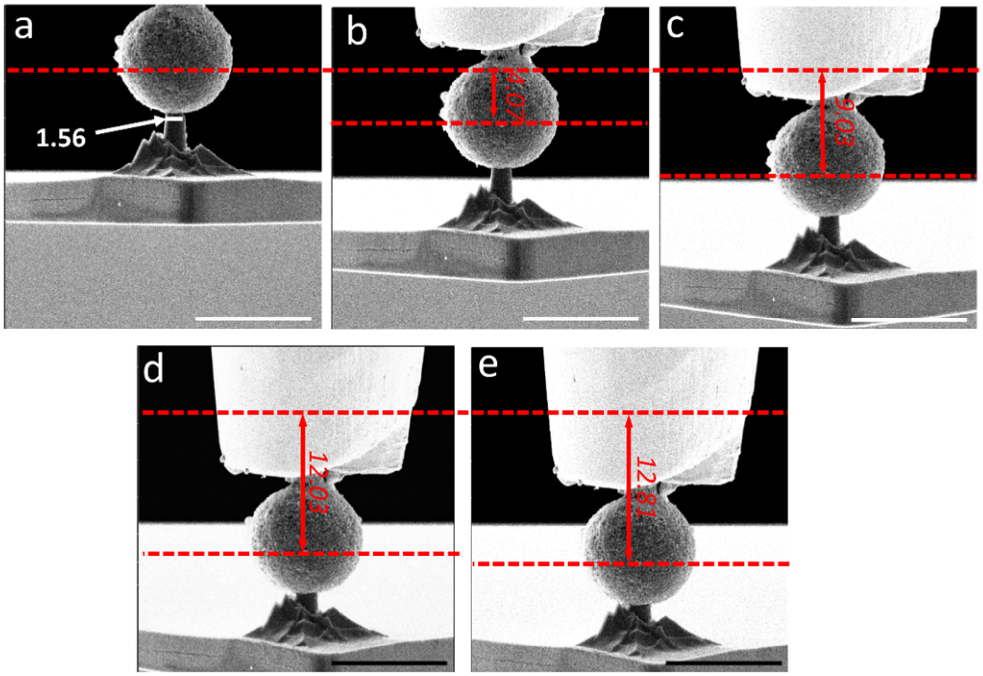

Supplementary Figure 31. Screenshots from another set of AFM-SEM experiment showing the AFM tip displacements/cantilever deflection from the beginning till the particle was penetrated by the AFM tip (scale bar = 10 μm). The unit of the numbers in the figures is μm.

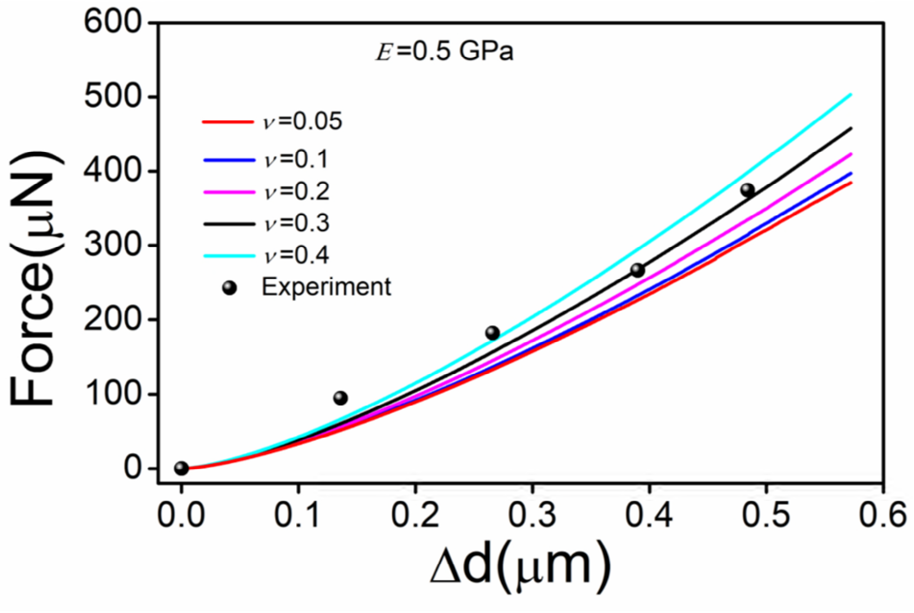

Supplementary Figure 32. Force versus deformation (Δd) of the microsphere from FE simulations with Young's modulus=0.5 GPa and different Poisson's ratios.

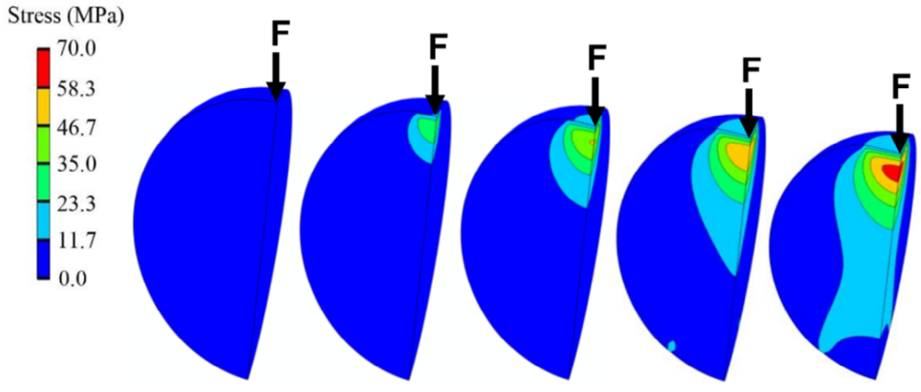

	Δd=0.0 μm	Δd=0.14 μm	Δd=0.27 μm	Δd=0.39 μm	Δd=0.48 μm
Max:	0.0 MPa	33.5 MPa	47.8 MPa	57.3 MPa	63.4 MPa
Min:	0.0 MPa	0.045 MPa	0.11 MPa	0.19 MPa	0.26 MPa

Supplementary Figure 33. The three-dimensional deformation process and von Mises stress distribution with the pressing of the microsphere particles for the Young's modulus 0.5GPa and the Poisson's ratio 0.3. The table shows the corresponding maximum/minimum stress values.

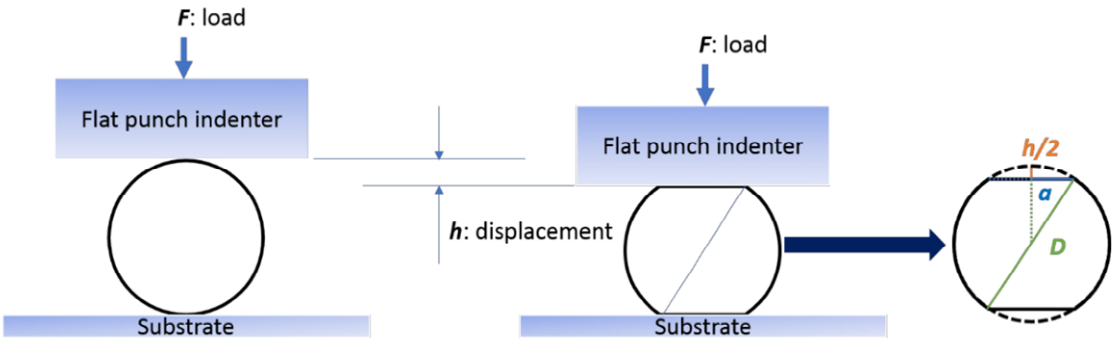

Supplementary Figure 34. Schematic of flat punch indentation of a CNT@Si@C sphere.

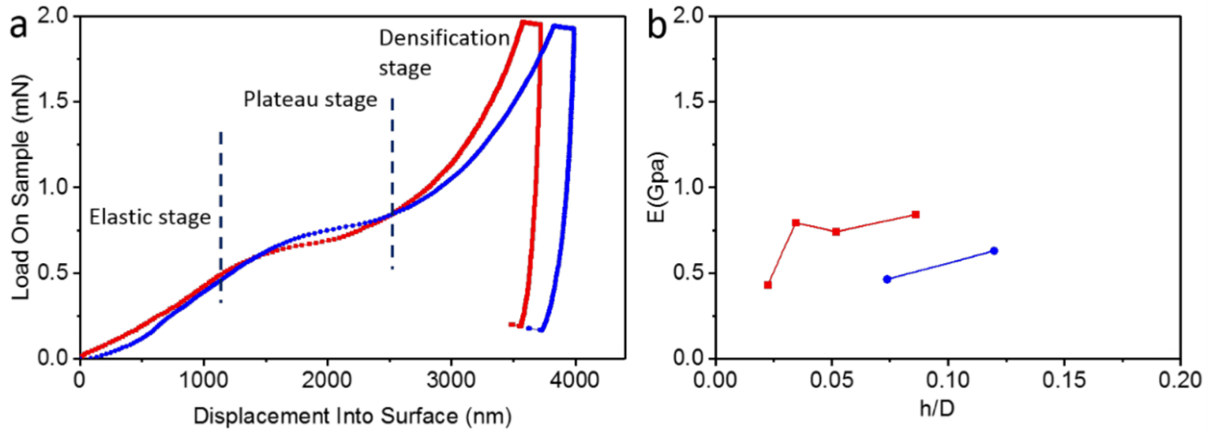

Supplementary Figure 35. **(a)** The curve of force vs particle (6.3±0.4 μm) displacement. **(b)** Elastic modulus of CNT@Si@C particle with the increase of displacement. These two colors correspond to two different particles.

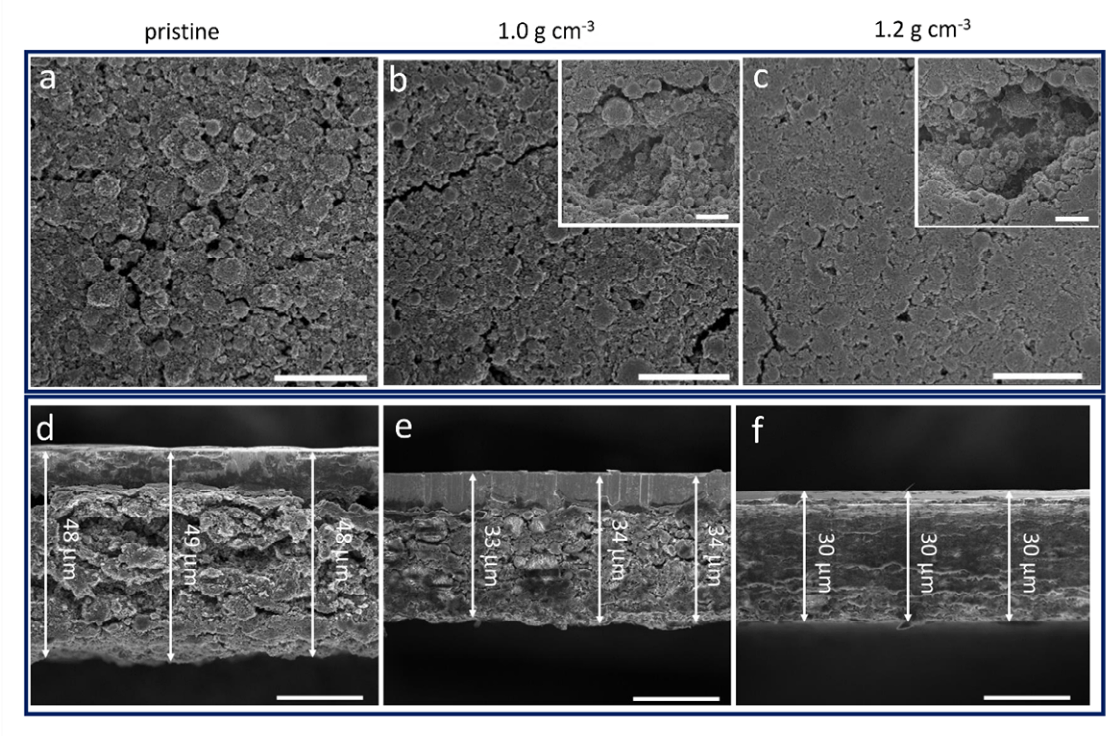

Supplementary Figure 36. SEM images of CNT@Si@C electrodes. (a-c) Top view and **(d-f)** cross-section SEM images of the pristine CNT@Si@C electrodes and electrodes calendered to 1.0 and 1.2 g cm^{-3}. Cu foil is 11 µm thick. Scale bar of Figure 36a-f is 20 µm, scale bar for inset Figures is 10 µm.

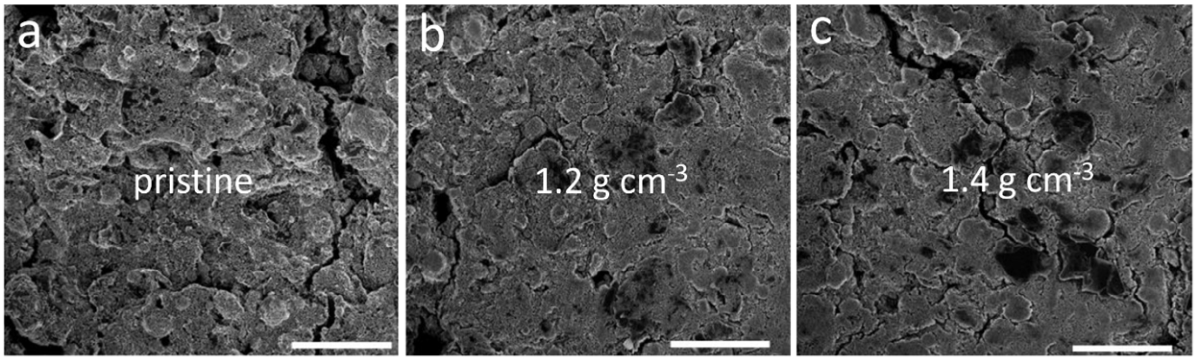

Supplementary Figure 37. SEM images showing the morphology of the CNT@Si@C-Gr electrodes with different calendering conditions. (a) Pristine electrode without calendering. **(b)** An electrode calendered to 1.2 g cm^{-3}. **(c)** An electrode calendered to 1.4 g cm^{-3}. Scale bar = 20 µm.

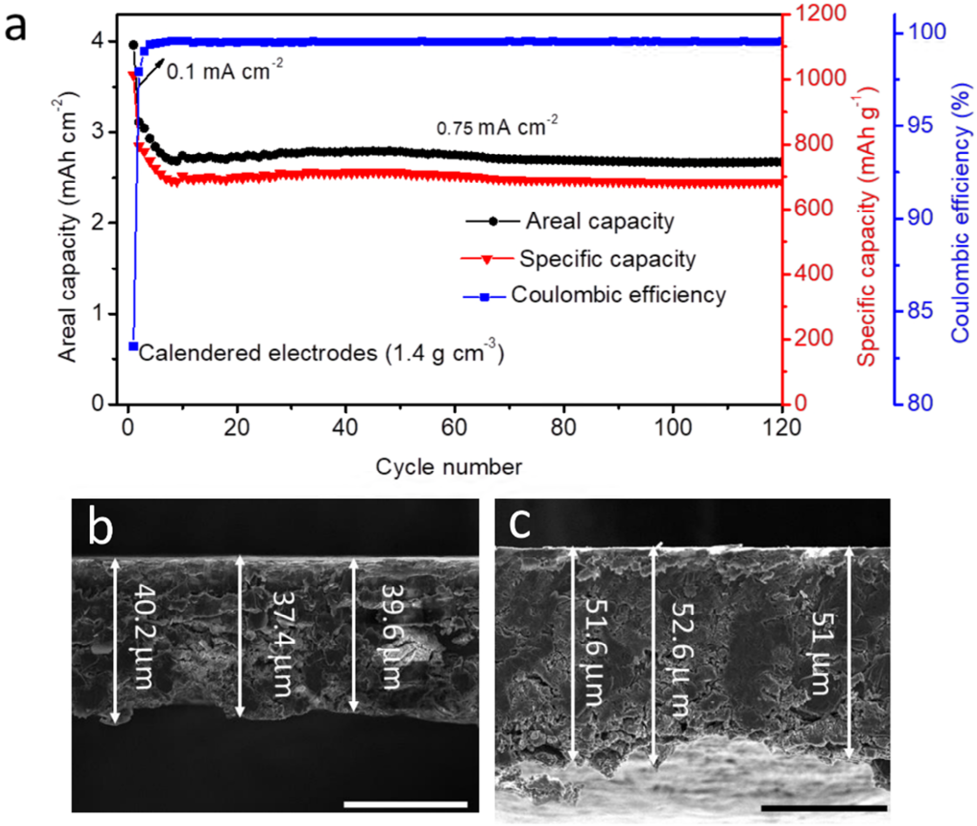

Supplementary Figure 38. Electrochemical performance of CNT@Si@C-Gr electrode with the density of 1.4 g cm^{-3}. **(a)** Long-term cycling performance of a typical CNT@Si@C-Gr electrode calendered to 1.4 g cm^{-3}. **(b)** Cross-section SEM image of the electrode before cycling (scale bar = 30 µm). **(c)** Cross-section SEM image of the electrode at full lithiation after 120 cycles (scale bar = 30 µm. Cu foil is 11 µm thick.

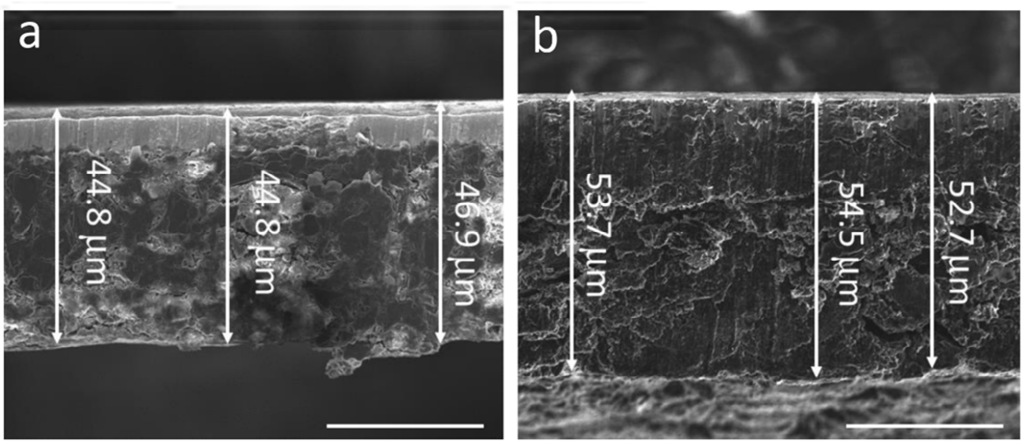

Supplementary Figure 39. SEM image of a calendared CNT@Si@C-Gr electrode (1.2g cc^{-1}). (a) before cycling (scale bar = 30 µm) and **(b)** after 1st lithiation (scale bar = 30 µm).

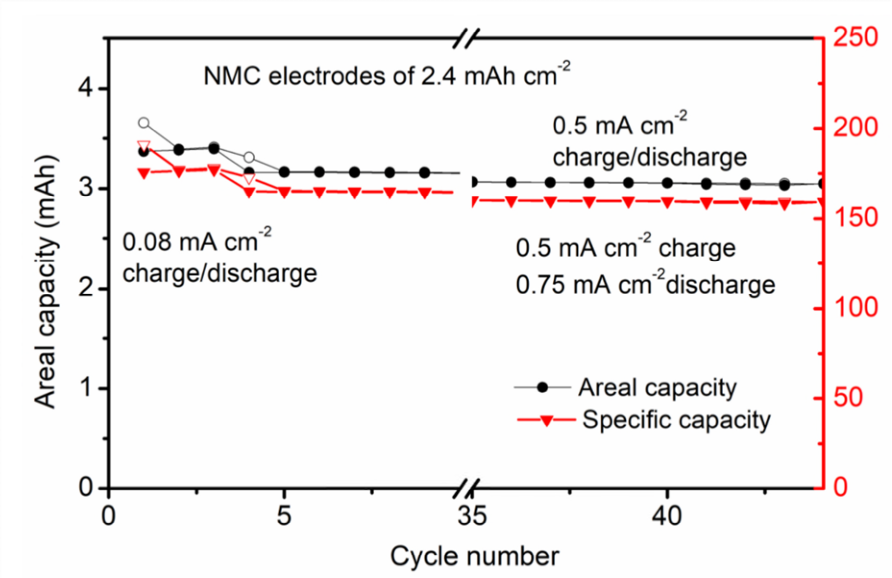

Supplementary Figure 40. Rate performance of a typical NMC333 cathode.

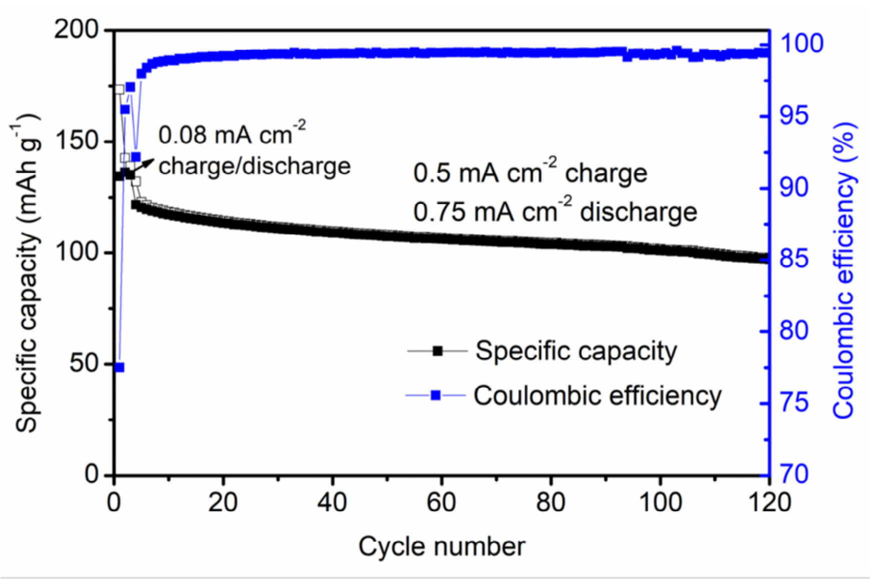

Supplementary Figure 41. Long-term cycling performance of full cells using NMC cathode and anode of CNT@Si@C-Gr without prelithiation.

Supplementary Tables

Supplementary Table 1. Surface area, pore volume and average pore size of CNT@SiO_2, CNT@Si and CNT@Si@C.

Sample	Surface area (m^2 g^{-1})	Pore volume (CC g^{-1})	Average pore size (nm)
CNT@SiO_2	77.25	0.5	26.25
CNT@Si	104.1	0.97	37.5
CNT@Si@C	61.5	0.43	20.0

Supplementary Table 2. Key parameters of the CNT@Si@C-Gr electrodes with and without calendering.

Sample	Electrode density	Electrode thickness	capacity density
	(g cc^{-1})	(μm)	(mAh cc^{-1})
Pristine (CNT@Si@C-Gr)	0.8	50.7	591
Calendered electrodes	1.2	33.8	844
	1.4	28.1	980

Supplementary Table 3. Comparison table of CNT@Si@C-Gr composite electrodes with other top works about silicon anodes.

	Half-cell					Full-cell
	Capacity retention	Coulombic efficiency	Volumetric capacity density	loading	swelling	
**This work**	92% @500@4.0 mg cm^{-2}	84%@1st cycle<br>99.9%@stable cycling	980mAh cc^{-1}@1.4g cc^{-1}<br>844 mAh cc^{-1}@ 1.2g cc^{-1}	3 mAh cm^{-2}	18.3%@ initial;<br>45%@120@1.4g cc^{-1};<br>34%@120@1.2g cc^{-1}	92%@500
**Si nanowire (Ref.12)**	10 cycles	73%@1st cycle;<br>90%@2nd cycle	Nanowire on stainless steel substrate			--
**DWSiNT (Ref. 15)**	88%@6000	76%@1st cycle;<br>99.9%@stable cycling	--	0.02-0.1mg cm^{-2}	--	--
**Pomegranate-liked Si@C (Ref.19)**	97%@1000@0.2 mg cm^{-2};<br>94%@100@ 3.12mg cm^{-2}	82%@ 1st cycle with no RF or charge to 2V;<br>99.87% @ stable cycling	310 mAh cc^{-1};<br>900-1270 mAh cc^{-1} after calendering	0.2mg cm^{-2}<br>~3 mAh cm^{-2}, on CNT paper	--	--
**Graphitic carbon shell@Si (Ref.23)**	85%@300	93.2%@ 1st cycle<br>99.9%@ stable cycling	--	0.8mg cm^{-2};	11%	90%@100
**Si nanolayer embedded Graphite (Ref.41)**	96%@100 with 517 mAh g^{-1}, 6% Si	92%@ 1st cycle<br>99.5%@ stable cycling	--<br>calculated to be ~827 mAh cc^{-1} (517mAh g^{-1}*1.6 g cc^{-1})	~3 mAh cm^{-2}	38%@50 cycles	92%@100
**Highly elastic binder(Ref.30)**	91%@150;<br>85%@370 with Li metal switched after 250	91.22%@1st cycle<br>99.6%@stable cycling	--	~3mAh cm^{-2};<br>1.04mg cm^{-2}	23% with 0.7 mg cm^{-2} loading	98%@50
**Ant-nest-like porous silicon(Ref.29)**	90%@1000@0.8 mg cm^{-2}	80.3%@ 1st cycle<br>99.9%@ stable cycling	1712mAh cc^{-1}@ 0.8g cc^{-1}	0.8-2.9 mg cm^{-2}	17-22.6%@ initial	84%@400

Supplementary Notes

Supplementary Note 1. Design of the CNT@SiO_2 core-shell coaxial cables (Supplementary Figure 3)

The thickness of SiO_2 coating layer was determined and optimized according to the capacity of the resulting material. The calculation can be illustrated as following:

$m (CNT)=\rho (CNT) * V (CNT)=1.7* \pi* h*r_2^2$

$m (SiO_2)= \rho (SiO_2) * V (SiO_2)=2.2* \pi* h* (r_3^2-r_2^2)$

r1= inner radius of the CNT, 4 nm

r2= outer radius of the CNT, 15 nm

r3= radius after the SiO_2 coating

ρ (MWNT)[1]=1.7 g cm^{-3}

ρ (SiO_2)[2]=2.2 g cm^{-3}

$m (SiO_2)/m(CNT)= 2.2* (r_3^2-r_2^2)/(1.7* r_2^2) =2.2*(r_3^2-15^2)/(1.7*15^2)$

The electrode specific capacity was set to 1000 mAh g^{-1}. When the active material is 80%, the specific capacity of the Si-based active material is 1250 mAh g^{-1}. That corresponds to 42 wt% Si in the composite assuming the specific capacity of Si is 3000 mAh/g. The weight percentage of Si in SiO_2 is 47 wt% (28/60), thus the amount of SiO_2 in the composite is 89 wt%.

$m (SiO_2) + m(CNT)=1$, so m(CNT)=11 wt%

Hence, $89/11 = 2.2*(r_3^2-15^2)/ (1.7*15^2)$, r_3 = 40.4nm. The SiO_2 thickness is 25.4 nm.

Supplementary Note 2. Raman spectroscopy of the CNT@Si and CNT@Si@@C composite (Supplementary Figure 14)

Raman spectroscopy of the CNT@Si and CNT@Si@@C composite provides a disorder-induced D band (~ 1330 cm^{-1}), graphite G band (~ 1580 cm^{-1}) and two-phonon scattering 2D band, similar to the literature [3]. After carbon coating, the intensities of D band and G band increased and G/D ratio is similar to the high quality carbon nanotube.

Supplementary Note 3. Fourier transform infrared (FTIR) spectroscopy spectra of CNT, CNT@Si and CNT@Si@C (Supplementary Figure 17)

Fourier transform infrared (FTIR) spectroscopy spectra of CNT, CNT@Si and CNT@Si@C was carried out to investigate the binding condition between Si and the CNTs. The intensity of the peaks at 3428 (O–H stretching), 2922 & 2855 (C–H asymmetric and symmetric stretching), 1572 (quinone groups), 1545 (C–OH bending) and 1381 cm^{-1} (C–O stretching) showed a decrease after thermite reduction, and the intensity of the peak at 1065-1091 cm^{-1} which corresponds to the characteristic vibrations of Si demonstrates an increase in CNT@Si. This is due to the bond formation between CNT and Silicon[4]. After coating, the broad peak at 1065-1091 cm^{-1} shows an intensity decrease and the peak is spitted into two sharp peaks, which suggests the formation of chemical bonding between Si and carbon[5].

Supplementary Note 4. Mechanical strength of CNT@Si@C (Supplementary Figure 29)

The mechanical strength of the CNT@Si@C particle can be calculated by measuring the AFM tip displacement/cantilever deflection until the particle cracks. The spring force constant of the AFM tip cantilever is 40 N/m. As shown in Supplementary Figure 29c, the AFM tip displacement/cantilever deflection before particle break is ~17.62 µm, thus the force can be calculated to be 705 µN (40*17.62).

As shown in Fig. S28c, the contact surface area is 3.9 μm^2. The pressure (P) can be calculated as following:

$$P = \frac{F}{S} = \frac{\frac{40nN}{nm} * 17{,}620\ nm}{\pi\gamma 2} = \frac{\frac{40nN}{nm} * 17{,}620\ nm}{3.90\ \mu m2} = 181MPa$$

Supplementary Note 5. Mechanical strength of CNT@Si@C (Supplementary Figure 31)

With the tip area of ~1.91 cm^2 and the AFM tip displacement/cantilever deflection of ~12.81 μm, the corresponding press can be calculated as following:

$$P = \frac{F}{S} = \frac{\frac{40nN}{nm} * 12{,}810\, nm}{\pi\gamma 2} = \frac{40\frac{nN}{nm} * 12{,}810\, nm}{1.91\mu m2} = 268\, MPa$$

Supplementary Note 6. Assessment of the Young's modulus by finite element (FE) simulations (Supplementary Figure 32-33)

To assess the Young's modulus of the particle, the particle deformation was simulated using the finite element commercial code (ABAQUS)[6]. In the simulations, a displacement constraint at the bottom of the particle and a pressing force on the top of the particle are applied. The radius of the spherical particle; the region of displacement constraint and the size of pressing tip all are set to be similar to that of the sample used in the *in situ* AFM-SEM measurement. A CAX4R finite element mesh type (deformable sample C04 and C1, 4-node, reduced-integration, axisymmetric, solid elements) with total 6000 elements is used. A parametric study by assigning the particle different Young's modulus (0.1, 0.5, 1.0, 2.0 and 5.0 GPa) and Poisson's ratios (0.05, 0.1, 0.2, 0.3 and 0.4) was carried out. The force-deformation curves were recorded in the simulations, and the results are plotted in Supplementary Figure 32. The force is the total force applied on the pressing tip. (Δd) denotes the deformation of the microsphere. Since the bottom of the particle is subject to displacement constraint where the displacement is zero, the (Δd) has the same meaning as shown in Figure 3a. The results show that 1) the force increases with the increase of both Young's modulus and Poisson's ratio for given deformation of the particle (Δd); 2) when Young's modulus and Poisson's ratio are 0.5GPa and 0.3, respectively, the curve of force vs deformation calculated from FE simulation is in good agreement with experimental data.

The shear deformation is the main deformation and failure mechanisms in a particle under uniaxial compression, the von Mises stress $\underline{\sigma}$ is defined as:

$$\sigma = \left\{ \frac{1}{2}\left[\left(\sigma_{11}-\sigma_{22}\right)^2 + \left(\sigma_{11}-\sigma_{33}\right)^2 + \left(\sigma_{22}-\sigma_{33}\right)^2 \right] + 3\left(\sigma_{12}^2+\sigma_{13}^2+\sigma_{23}^2\right) \right\}^{0.5}.$$

where σ_{ij} is the stress component. The von Mises stress can well describe the local shear deformation. Supplementary Figure 33 shows the distributions of the von Mises stress on two cross sections of the particle at different deformation stages (Δd). It can be seen that the von Mises stress beneath the pressing tip is much larger than that near the bottom of the particle where a displacement constraint is applied. The large von Mises stress may cause shear fracture. The Supplementary Movies S5 and S6 shows that shear failure.

Supplementary Note 7. Schematic of flat punch indentation of a CNT@Si@C sphere (Supplementary Figure 34-35)

h: displacement
a: contact radius
D: particle diameter

$$a = \frac{\sqrt{D^2 - (D-h)^2}}{2}$$

The set-up of flat punch indentation is illustrated in Supplementary Figure 34. As shown in Supplementary Figure 35a, the displacement and the force at the end of elastic stage are 1, 079 nm and 0.47 mN, respectively. Thus, the pressure (P) can be calculated as following:

Contact radius $a = \frac{\sqrt{6.3^2-(6.3-1.079)^2}}{2}$= 1.76 μm

$P = \frac{F}{S} = \frac{0.47mN*1000}{\pi a2} = \frac{470\ \mu N}{9.72\ \mu m2} = 48.4MPa$ (the stress at the end of elastic deformation)

The displacement and the force at the end of elastic stage are 3, 500 nm and 1.96 mN, respectively. Thus, the pressure (P) can be calculated as following:

Contact radius $a = \frac{\sqrt{6.3^2-(6.3-3.5)^2}}{2}$= 2.62 μm

$P = \frac{F}{S} = \frac{1.96mN*1000}{\pi a2} = \frac{1960\ \mu N}{21.5\ \mu m2} = 91.2\ MPa$ (the stress at the end of densification)

Elastic modulus calculation:

1) Stiffness, S (determined by the initial 20% of the unloading)

$$S=dF/dh$$

2) Reduced modulus, Er

$$E_r=S/2a$$

3) The elastic modulus of the test sample, E, is the determined form the E_r:

$$\frac{1}{E_r}=\frac{1-v^2}{E}+\frac{1-{v_i}^2}{E_i}$$

A rough estimate of v=0.3 is used as Poisson's ratio of the test material; for the diamond indenter, E_i =1141 GPa and $v_i = 0.07$. According to the above calculation formulas and the force curve vs. displacement (Figure S35a), the elastic modulus of the particle vs displacement can be plotted as Supplementary Figure 35b. When the displacement at the end of elastic stage is 1,079 nm, h/D is around 0.18 and elastic modulus is ~0.5~0.9 GPa.

Supplementary Methods

CNT surface functionalization: the CNTs were wrapped with polymer (polyvinylpyrrolidone, PVP) so that the modified CNTs can be easily dispersed in polar solvents such as water. In a typical experiment, CNTs (110 mg) were dispersed in a 0.5 wt% PVP (with a molecular weight of 40,000) water solution (200 mL) and sonicated for 2 hr. Then the homogeneous CNT solution was stirred for 12 hr. Finally, the PVP-wrapped CNT material (PVP-CNT) was obtained by vacuum filtration through a 0.1 μm polyvinylidene fluoride (PVDF) membrane filter.

Synthesis of CNT@SiO$_2$: in a typical experiment, 110 mg of PVP-CNT was dispersed into a solution of 160 mL ethanol and 16 mL H_2O and sonicated to form a homogeneous solution. Then, 2 mL NH_3•H_2O (25 wt%) and 3.16 mL tetraethoxysilane (TEOS) were added into the above dispersion under stirring for 24 hr. The product was filtered and washed with ethanol and deionized water several times, and was redisposed in 36 mL water.

Microemulsion-based assembly of CNT@SiO$_2$ into microspheres: the obtained water dispersion (2 mL) was mixed with 16 mL 1-octadecene (ODE) solution containing 0.3 wt% of emulsion stabilizer and homogenized at 8000 rpm for 2 min. The mixture was then heated at 95~98 °C for 4 hr. After evaporation of water, the CNT@SiO$_2$ microspheres were collected by centrifugation, and washed with petroleum ether once. The final powder was calcined at 550 °C for 1 hr in argon to remove the organics and condense the CNT@SiO$_2$.

Preparation of CNT@Si: CNT@Si was prepared via a modified aluminothermic reaction with $AlCl_3$ as the molten salt. CNT@SiO$_2$ was mixed with $AlCl_3$ and Al metal at the ratio of ~1:6:1.6 (weight ratio). The mixture was sealed in a Swagelok® reactor and heated in a tube furnace under an Ar atmosphere at 350 °C for 15 hr. After cooling to room temperature, the obtained products were first immersed in H_2O and subsequently in 1 M HCl to remove by-products. Finally, the products were collected, washed with H_2O and ethanol, and vacuum-dried at 60 °C overnight.

Fabrication of the carbon coated CNT@Si composite: The CNT@Si was coated with carbon via a CVD method using acetylene as the carbon source. The CVD process was carried out at 700 °C for 30 min under a mixture of argon and acetylene gas[7]. After coating, the furnace was cooled down naturally in an inert atmosphere.

Structure characterization: XRD was carried out on a Rigaku MiniFlex II diffractometer with Cu Kα radiation. Raman spectra were taken on a Bruker optic SENTERRA (R-200L) Raman spectrometer with a laser wavelength of 633 nm. Thermogravimetric analysis (TGA) measurement was carried out in oxygen atmosphere in the temperature range from 30 to 800 °C with a heating rate of 5 $°Cmin^{-1}$. Particle size distribution was performed by high definition digital particle size analyzer (Micromeritics Saturn Digisizer II). Fourier transform infrared spectroscopy (FTIR) was carried out on VERTEX (Bruker). XPS analysis was performed on a Physical Electronic Quantera scanning X-ray microprobe with a focused monochromatic Al K_a X-ray source.

In situ TEM characterization: Volume expansion of CNT@Si and CNT@Si@C microspheres upon lithiation was checked by a solid-state "nano-battery" configuration using a Nanofactory biasing holder inside a FEI Titan S/TEM. The microspheres were dry suspended on a Pt rod and brought into contact with another W probe covered with Li/Li_2O. The naturally formed Li_2O on Li serves as a solid electrolyte. The lithiation was enabled by applying a negative bias (-2 V) to the Pt end. The full lithiation was confirmed by prolonged lithiation after the maximum volume expansion is reached.

Electrochemical tests: The CNT@Si and CNT@Si@C electrodes were prepared by mixing 80 wt% CNT@Si@C material, 5 wt% Super P and 15 wt% dreambond® polyimide binder to form a slurry, which was then pasted on a Cu foil. The CNT@Si@C-Gr composite electrodes were composed of 30 wt% CNT@Si@C and 58wt% graphite, 2wt% Super p and 10 wt% polyacrylic acid binder. The $Li(Ni_{1/3}Mn_{1/3}Co_{1/3})O_2$ (NMC) electrode was prepared by mixing NMC, Super P, and polyvinylidene difluoride dissolved in N-methyl-2-pyrrolidone in a ratio of 96 : 2: 2 to form a slurry, which was then pasted on an Al foil. After vacuum drying, the electrodes were tested in coin cells with Li metal as the counter and reference electrodes. The electrolyte was 1 M $LiPF_6$ in a mixture of ethylene carbonate and diethyl carbonate (3:7, w/w), plus 10 wt% fluoroethylene carbonate. The CR2032-type coin cells were assembled in an argon-filled glovebox with Celgard 2500 as separator. The electrochemical performance was evaluated using galvanostatic charge–discharge protocols on an Arbin BT-2000 battery system at 30 °C. In the case of Si-based electrodes, the cutoff voltage was 0.02 V vs. Li/Li^+ for lithiation and 1.5 V vs. Li/Li^+ for delithiation. The specific capacity was calculated based on the total weight of the

active composite material. Electrode thickness change during cycling was measured using an electrochemical dilatometer (EL-Cell, Germany). For NMC cathode, the cut-off voltage was 2.7-4.3 V and the electrolyte is the same as that used for Si-based anodes. The full cells were tested between 2.8 and 4.2 V with constant current protocol. The full cell specific capacity was calculated based on cathode. CNT@Si@C||NMC333 and CNT@Si@C-Gr||NMC full-cells are evaluated under the same condition.

Supplementary References

1 Lu, Q. *et al.* Determination of carbon nanotube density by gradient sedimentation. *J. Phys. Chem. B* **110**, 24371-24376, (2006).

2 Van Hoang, V. Molecular dynamics simulation of amorphous siO(2) nanoparticles. *J. Phys. Chem. B* **111**, 12649-12656, (2007).

3 Chen, J. *et al.* Carbon nanotube network modified carbon fibre paper for Li-ion batteries. *Energy Environ. Sci.* **2**, 393-396, (2009).

4 Yaghoubi, A. & Alavi Nikje, M. M. Silanization of multi-walled carbon nanotubes and the study of its effects on the properties of polyurethane rigid foam nanocomposites. *Composites Part A: Applied Science and Manufacturing* **109**, 338-344, (2018).

5 An, W. *et al.* Scalable synthesis of ant-nest-like bulk porous silicon for high-performance lithium-ion battery anodes. *Nat. Commun.* **10**, 1447, (2019).

6 Ahmed, S. *et al.* Investigation of affecting parameters on the effective modulus and natural frequency of wavy carbon nanotubes. *Journal of Physics and Chemistry of Solids* **121**, 121-127, (2018).

7 Li, X. L. *et al.* Mesoporous silicon sponge as an anti-pulverization structure for high-performance lithium-ion battery anodes. *Nat. Commun.* **5**, 7, (2014).
