# Supplementary material for: Hierarchical porous silicon structures with extraordinary mechanical strength as high-performance lithium-ion battery anodes
Source: Nat Commun. 2020 Mar 19;11:1474. doi: 10.1038/s41467-020-15217-9 (PMC7081208; doi:10.1038/s41467-020-15217-9)
Supplement: Supplementary file 2 — Description of Additional Supplementary Files [file 41467_2020_15217_MOESM2_ESM.pdf]

# Description of Additional Supplementary Files

**Supplementary Movie 1.** Lithiation of the CNT@Si microsphere shown in Supplementary Figure 17.

**Supplementary Movie 2.** Lithiation of the CNT@Si microsphere shown in Supplementary Figure 17.

**Supplementary Movie 3.** Lithiation of the CNT@Si@C microsphere shown in Figure 2.

**Supplementary Movie 4.** Lithiation of CNT@Si@C cable at different cycles shown in Supplementary Figure 19.

**Supplementary Movie 5.** In situ AFM-SEM experiment of the CNT@Si@C microsphere shown in Figure 3 and Supplementary Figure 19-20.

**Supplementary Movie 6.** In situ AFM-SEM experiment of the CNT@Si@C microsphere shown in Supplementary Figure 21.

**Supplementary Movie 7.** FE simulation results with the Young's modulus 0.5GPa and the Poisson's ratio 0.3 showing the deformation process and von Mise stress evolution for the CNT@Si@C microsphere.
